# Linking Altered Flow Regimes to Biological Condition: an Example Using Benthic Macroinvertebrates in Small Streams of the Chesapeake Bay Watershed

**DOI:** 10.1007/s00267-021-01450-5

**Published:** 2021-03-12

**Authors:** Kelly Oliver Maloney, Daren Milo Carlisle, Claire Buchanan, Jennifer Lynn Rapp, Samuel Hess Austin, Matthew Joseph Cashman, John André Young

**Affiliations:** 1 U.S. Geological Survey, Eastern Ecological Science Center, Kearneysville, WV USA; 2 U.S. Geological Survey, Lawrence, KS USA; 3 Interstate Commission on the Potomac River Basin (ICPRB), Rockville, MD USA; 4 U.S. Geological Survey, Virginia and West Virginia Water Science Center, Richmond, VA USA; 5 U.S. Geological Survey, Maryland-Delaware-District of Columbia Water Science Center, Baltimore, MD USA

**Keywords:** Ecological flows, Odds ratios, Hydrologic metrics, Benthic macroinvertebrates, Altered flows, Hydrologic alteration

## Abstract

Regionally scaled assessments of hydrologic alteration for small streams and its effects on freshwater taxa are often inhibited by a low number of stream gages. To overcome this limitation, we paired modeled estimates of hydrologic alteration to a benthic macroinvertebrate index of biotic integrity data for 4522 stream reaches across the Chesapeake Bay watershed. Using separate random-forest models, we predicted flow status (inflated, diminished, or indeterminant) for 12 published hydrologic metrics (HMs) that characterize the main components of flow regimes. We used these models to predict each HM status for each stream reach in the watershed, and linked predictions to macroinvertebrate condition samples collected from streams with drainage areas less than 200 km^2^. Flow alteration was calculated as the number of HMs with inflated or diminished status and ranged from 0 (no HM inflated or diminished) to 12 (all 12 HMs inflated or diminished). When focused solely on the stream condition and flow-alteration relationship, degraded macroinvertebrate condition was, depending on the number of HMs used, 3.8–4.7 times more likely in a flow-altered site; this likelihood was over twofold higher in the urban-focused dataset (8.7–10.8), and was never significant in the agriculture-focused dataset. Logistic regression analysis using the entire dataset showed for every unit increase in flow-alteration intensity, the odds of a degraded condition increased 3.7%. Our results provide an indication of whether altered streamflow is a possible driver of degraded biological conditions, information that could help managers prioritize management actions and lead to more effective restoration efforts.

## Introduction

Anthropogenic activities have drastically altered the natural flow regime of most of the world’s rivers (Nilsson et al. 2005; Poff et al. 2007), which has important consequences for associated biota. Flow drives multiple in-stream physical, chemical, and thermal variables (Power et al. 1995; Poff et al. 1997; Bunn and Arthington 2002) that together comprise the habitat template. Understanding how altered flows affect these habitat variables and biological communities has been a focus of study for many years (Poff and Zimmerman 2010) and is crucial for improving conservation and restoration of these systems, especially given projected future changes to landscapes and climate and their potential effects on stream systems (Pyne and Poff 2017; Maloney et al. 2020). However, identifying an effect of altered flows on aquatic communities is complicated by the myriad of factors simultaneously influencing these communities, often through nonlinear and interactive pathways (Matthaei et al. 2010; Buchanan et al. 2013; Schmidt et al. 2019).

An optimal approach to assess the effects of altered flow regimes on biological communities is to have paired long-term observed hydrological timeseries and biological data where the effects of confounding stressors (e.g., sedimentation) are minimal or accounted for. Here, hydrologic metrics (HMs) that characterize components of the flow regime (e.g., duration, frequency, magnitude, timing, and rate of change, Poff et al. 1997) are directly calculated from observed hydrologic timeseries (e.g., Richter et al. 1996), then compared before/after a known alteration (Pegg et al. 2003), or related to natural hydrologic expectations. Studies with such paired datasets have revealed ecological responses to various types and severities of streamflow alteration at regional and national scales (Carlisle et al. 2011, 2019; Klaar et al. 2014; Buchanan et al. 2017). However, in many cases, paired biological and historical hydrological timeseries data are limited, which inhibits compilation of sufficiently large datasets for rigorous analysis, especially within specific river basins. To overcome this limitation, researchers often use one of two approaches to model streamflow for reaches that have a biological sample but no observed flow data (Poff et al. 2010). First, rainfall-runoff simulation models are used to synthesize the daily hydrograph, from which HMs are subsequently calculated (Kennen et al. 2008; Phelan et al. 2017; Moltz et al. 2018). Second, statistical models of HMs are typically calibrated over large regions (e.g., Carlisle et al. 2010; Eng et al. 2013, 2017; Klaar et al. 2014; Patrick and Yuan 2017), and subsequently applied to predict the HM rather than the daily hydrograph. Each method has its benefits and limitations. For example, simulation models provide flow data from which different HMs can be calculated, but may require added effort to calibrate. In contrast, statistical modeling approaches are relatively simple to develop but require a separate model for every HM—which can become cumbersome if many HMs are considered. Further, recent evidence suggests that, compared to statistical models, simulation models may display pronounced positive or negative bias in predicting several ecologically relevant streamflow characteristics (Murphy et al. 2013). For some management and research questions, however, it may be sufficient to simply predict whether or not an HM is modified from natural expectations in an ungaged stream rather than the actual numeric value of the HM, which potentially simplifies the performance requirements of the statistical models (Knight et al. 2014).

Small streams (e.g., <4th order), which comprise the bulk of stream reaches and length (Leopold et al. 1964), are especially challenging for ecohydrological studies. First, these systems are more sparsely gaged than larger-order streams, which limits paired biological and hydrological observations, especially within single river basins (Dunbar et al. 2010; Deweber et al. 2014). Moreover, compared to larger-order streams, headwater systems are more tightly coupled with hillslope processes and have more temporal and spatial variation (Gomi et al. 2002). Small streams also have different biological communities than larger systems (Vannote et al. 1980; Riley et al. 2018), and may be more susceptible to flow alteration, especially related to withdrawals (Rapp et al. 2020). As a result, a comprehensive modeling of small streams is necessary to develop inferences on the relationship between altered flows and biological communities.

Benthic macroinvertebrates are a key assemblage in all running waters. Not only do they provide essential functions such as nutrient and energy transfer through food webs (Covich et al. 1999), but they are also a very diverse assemblage with a variety of life histories and tolerances to external stressors (Meyer et al. 2007). Benthic macroinvertebrates have been shown to be negatively affected by anthropogenically altered landscapes (e.g., urbanization and agriculture, King et al. 2005; Walsh et al. 2005; Maloney et al. 2018) and altered streamflows (Kennen et al. 2008; Bruno et al. 2016; Phelan et al. 2017). Because of these characteristics, benthic macroinvertebrates are key bioindicators in stream monitoring programs around the world (Bonada et al. 2006; Carter et al. 2017) and are an excellent indicator to assess stream degradation, i.e., difference from reference expectations, associated with altered flows. However, partitioning out the effects of a single stressor, such as altered flows, is complicated by the fact that benthic macroinvertebrate assemblages are influenced by the cumulative effects of multiple stressors, only some of which may be related to altered streamflow.

Here, our goal was to evaluate the potential of predicted altered flow regimes to explain degraded macroinvertebrate communities in streams of the Chesapeake Bay watershed, a naturally and anthropogenically diverse landscape in the mid-Atlantic region of the United States. Our primary hypothesis was that macroinvertebrate communities are more likely to be degraded in stream reaches that are predicted to be hydrologically altered relative to reaches without hydrological alteration—across all types of land-use settings. In addition, the large sample size (*n* = 4522) enabled us to evaluate whether the likelihood of degraded macroinvertebrate communities in flow-altered reaches would differ between different land-use settings. We also hypothesized that the likelihood of degraded macroinvertebrate communities would increase with the predicted intensity of flow alteration. Finally, we used a set (*n* = 50) of paired gaged and macroinvertebrate sampling sites to compare the relationship between observed and modeled estimates of hydrologic alteration with stream condition.

## Methods

### Study Area

**Fig. 1:**
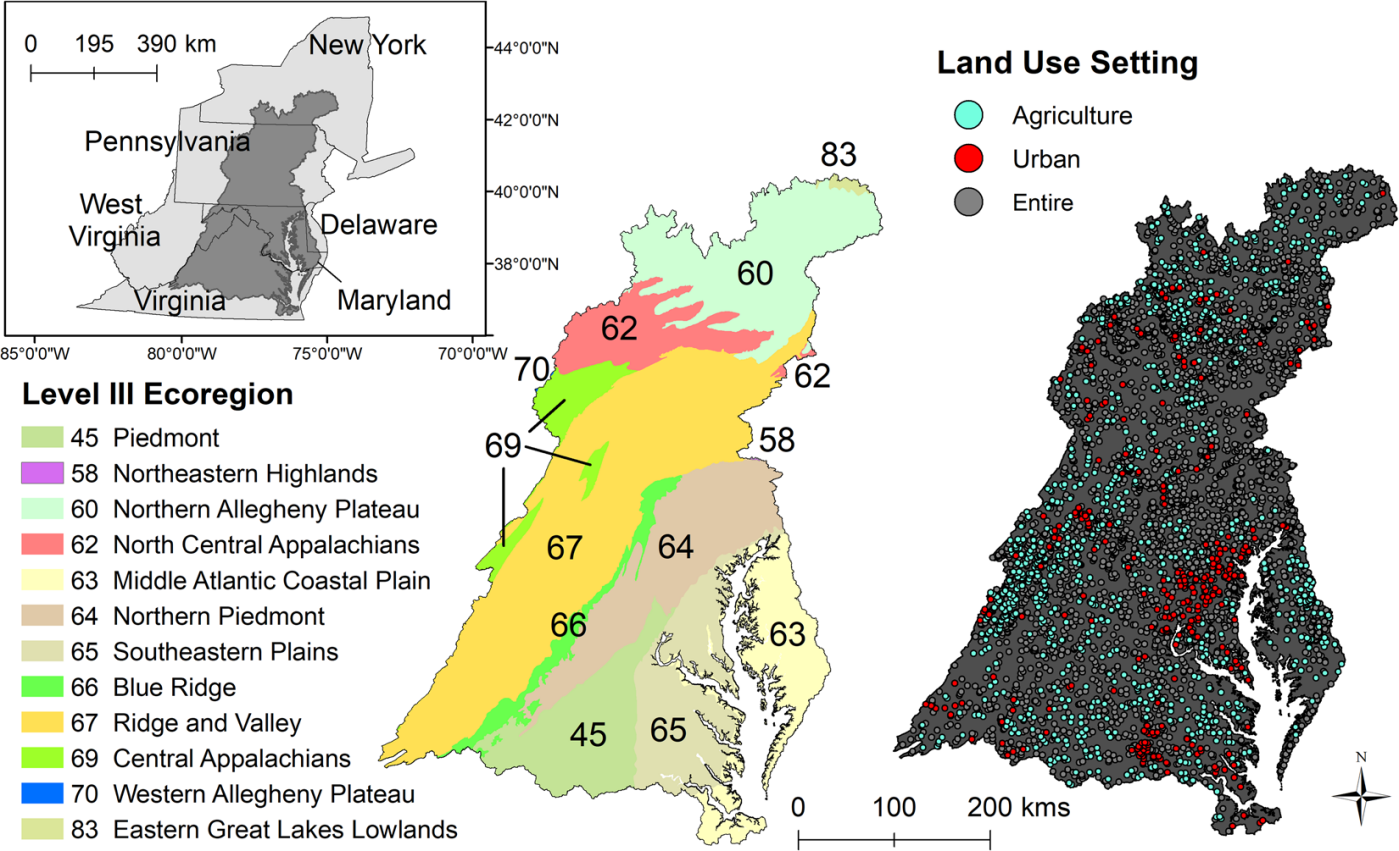
Map of Chesapeake Bay watershed highlighting the Level III ecoregions (left) and Chesapeake Bay Basin-wide Index of Biotic Integrity (Chessie BIBI) sites by land-use setting (right). The inset show the Chesapeake Bay watershed in relation to the mid-Atlantic states of the United States

The Chesapeake Bay watershed is in the mid-Atlantic portion of the United States and drains approximately 168,000 km^2^ of Delaware, Maryland, New York, Pennsylvania, Virginia, West Virginia, and the District of Columbia (Fig. 1). The watershed includes several major river basins (the Susquehanna, Potomac, James, Rappahannock, and York) that drain into the Chesapeake Bay, the largest estuary in the United States (Chesapeake Bay Program 2017). Currently the watershed supports a population of over 18 million people and includes the major cities Baltimore, Maryland; Washington, DC; Harrisburg, Pennsylvania; Richmond, Virginia. In 2011, agricultural land cover comprised 23.8% of the watershed and urban/suburban (hereafter “urban”) comprised 10.8% (2011 National Land Cover Database, NLCD, Homer et al. 2015). Restoration of the bay and watershed is led and directed by the Chesapeake Bay Program, which is a partnership of federal and state agencies, local government, nonprofit organizations, and academic institutions. One goal of this program is to improve stream health and function for 10% of stream miles above a 2008 baseline (Chesapeake Bay Program 2017). Understanding the spatial distribution and magnitude of hydrological alteration in the watershed may help identify possible reaches where the extent of altered flows may be driving stream biological degradation, which can then be used to identify more effective restoration options to meet this program goal.

### HMs and Alteration

We used data from Eng et al. (2019) who published quantitative and categorical estimates of hydrologic alteration for each of twelve HMs (Table 1) for 3355 U.S. Geological Survey (USGS) gages across the contiguous United States. Observed (*O*_hm_) metric values were calculated from daily flow data (1980–2014) from each gage. Briefly, a machine-learning model was developed to predict the expected natural value of each HM (*E*_hm_) using natural landscape variables (e.g., elevation, geology, and soils) and climatic conditions matched to observed flows at 848 USGS gaging stations in hydrologically least disturbed (sensu Stoddard et al. 2006) watersheds (Falcone et al. 2010). Models were then subsequently applied to 3355 USGS gages in watersheds with known human influences on hydrology for the purpose of estimating the natural baseline (*E*_hm_) for the same time period (1980–2014). For each of these gages, the ratio *O*_hm_/*E*_hm_ was used to categorize hydrologic alteration as “indeterminant” (*O*_hm_/*E*_hm_ within the range of site-specific model error), “inflated” (*O*_hm_/*E*_hm_ > 1 and outside of model error), or “diminished” (*O*_hm_/*E*_hm_ < 1 and outside of model error) (see Eng et al. 2019 for additional detail).

**Table 1:** Hydrologic metrics (HMs) codes and descriptions from Eng et al. (2019) and used in study

Code	Hydrologic metric	Description
HF_DUR	High-flow duration	Average annual duration (days) of flow pulses > the 90th percentile of daily flows
HF_FRE	High-flow frequency	Average annual number of flow pulses > the 90th percentile of daily flows
HF_MAG	High-flow magnitude	Average of annual daily flows > the 99th percentile of daily flows
HF_SEA	High-flow timing/seasonality	Seasonal distribution of flows > the 90th percentile of daily flows
HF_VAR	High-flow variability	CV of the annual maximum of daily flows
LF_DUR	Low-flow duration	Average annual duration (days) of flow pulses < the 10th percentile of daily flows
LF_FRE	Low-flow frequency	Average annual number of flow pulses < the 10th percentile of daily flows
LF_MAG	Low-flow magnitude	Average of annual daily flows < the 10th percentile of daily flows
LF_SEA	Low-flow timing/seasonality	Seasonal distribution of flows < the 10th percentile of daily flows
LF_VAR	Low-flow variability	CV of the annual minimum of daily flows
SKEW	Skew	Average annual skew of daily flows
RISES	Daily rises	Number of days where daily flow > previous day/total number of days

Percentiles of daily flows are defined as long-term (20+ years) natural expected values based on models from Eng et al. (2019). Skew was computed as the third moment of the daily flows

*CV* coefficient of variation

Here, we used a subset of 1235 gages that were located in four aggregated Level III ecoregions (U.S. Environmental Protection Agency 2013) used in Eng et al. (2019) within the Chesapeake Bay watershed (Eastern Highlands, *n* = 354; Northeast/Mixed Wood Shield, *n* = 367; Southeast Coastal Plains, *n* = 59; Southeast Plains, *n* = 455, Table 2). Upstream drainage area ranged from very small (5.0–9.4 km^2^) to very large (2,908.3–49,802.3 km^2^) and gages were most frequently located on streams draining catchments over 100 km^2^ (Fig. S1). We built random-forest models (Breiman 2001) for each HM that predict the hydrologic alteration category using drainage area and previously summarized upstream catchment accumulated values (NHDPlus v2.1, 1:100,000 scale) for 15 landscape variables that describe anthropogenic stress related to urban development, agriculture, water usage, and augmentation (Table S1, Wieczorek et al. 2018). For each HM, we used a random 75% subset (*n* = 926) as a training dataset and 25% (*n* = 309) as an independent test dataset. Random-forest models were optimized using the tuning function and balanced to the lowest classification size using the sampsize function in the randomForest package (Liaw 2002, Table 3). Accuracy of each model was evaluated with area under the receiver operation characteristic curve (AUC) with the AUC function in the HandTill2001 R package that extends AUC to work with multiple categories (Cullmann 2019), and accuracy, kappa, sensitivity (true positive rate), and specificity (true negative rate) with the confusionMatrix function from the caret R package (Kuhn 2020). AUC values range from 1 (perfect model prediction) to 0 (reciprocal model prediction) and values >0.5 indicate that model performance was better than would be expected from chance alone (Swets 1988; Hirzel et al. 2006). Kappa infers agreement between predicted and observed data where values <0.00 indicate poor agreement, 0.00–0.20 slight agreement, 0.21–0.40 fair agreement, 0.41–0.60 moderate agreement, 0.61–0.8 substantial agreement, and 0.81–1.00 almost perfect agreement (Landis and Koch 1977). We then used these optimized models to predict (i.e., extrapolate) hydrologic alteration category for all Chesapeake Bay watershed stream reaches in the NHDPlus v2.1 dataset and examined these predictions for any noticeable spatial patterns. For the predictor variables, means and medians of the gaged and Chesapeake watershed datasets closely aligned; however, the gaged dataset often did not capture the maximum values in the Chesapeake watershed (Table S1). Thus, across the Chesapeake Bay watershed, we have confidence in our model predictions for all catchments, except those with extreme (near maximum range) landscape conditions.

**Table 2:** Drainage area (km^2^) summary statistics for gages used in hydrologic metric calculations by aggregated Level III ecoregion used in Eng et al. (2019)

Aggregated Level III ecoregion	*N*	Mean	Median	Minimum	Maximum
Eastern Highlands	354	2044.4	552.2	5.0	29,952.1
Northeast/Mixed Wood Shield	367	1837.3	340.9	9.4	47,364.2
Southeast Coastal Plains	59	201.0	94.3	5.4	2908.3
Southeast Plains	455	3016.8	617.6	5.0	49,802.3

**Table 3:** Model performance statistics and tuning parameters (mtry, n) for random-forest models predicting alteration of each hydrologic metric

							Sensitivity			Specificity		
Metric	mtry	*n*	OOB	AUC	Accuracy	Kappa	Diminished	Indeterminant	Inflated	Diminished	Indeterminant	Inflated
HF_DUR	5	257	27.11	0.86	0.74	0.60	0.68	0.77	0.76	0.90	0.85	0.85
HF_FRE	3	252	31.21	0.84	0.69	0.52	0.78	0.71	0.54	0.79	0.81	0.92
HF_MAG	4	154	29.37	0.80	0.68	0.47	0.75	0.65	0.49	0.77	0.85	0.87
HF_SEA	3	270	33.80	0.82	0.64	0.46	0.67	0.62	0.64	0.82	0.80	0.84
HF_VAR	6	170	35.21	0.75	0.60	***0.37***	0.66	0.63	***0.39***	0.74	0.82	0.82
LF_DUR	4	126	34.53	0.82	0.68	0.48	0.68	0.72	0.56	0.83	0.85	0.83
LF_FRE	4	152	34.13	0.78	0.67	0.47	***0.40***	0.69	0.77	0.85	0.87	0.77
LF_MAG	5	177	33.59	0.82	0.67	0.48	0.49	0.73	0.71	0.87	0.84	0.77
LF_SEA	5	187	35.21	0.81	0.67	0.49	0.80	0.70	0.41	0.78	0.84	0.87
LF_VAR	7	109	35.78	0.78	0.69	0.48	0.77	0.72	0.31	0.78	0.84	0.88
SKEW	3	151	30.35	0.81	0.68	0.47	0.73	0.66	0.50	0.79	0.81	0.89
RISES	3	77	31.43	0.79	0.69	0.45	***0.28***	0.70	0.75	0.91	0.82	0.74

Bold and italicized indicate a kappa < 0.41 or a sensitivity or specificity score < 0.40

*mtry* the optimal number of variables randomly sampled as predictors at each split identified during model tuning for random-forest models, *n* sample size used in random-forest models to balanced analysis, *OOB* out of bag error rate from models, *AUC* area under the receiver operation curve

### Benthic Macroinvertebrates

We used data from the Chesapeake Bay Basin-wide Index of Biotic Integrity (Chessie BIBI, Smith et al. 2017) version 2.02, which contains benthic macroinvertebrate data from 21,288 sampling events collected by 28 state, county, regional, federal, and other monitoring programs from 1992 to 2015. The dataset focused on first- to fourth-order streams (1:100,000 scale NHDPlus v2.1), excluded data collected in December, January, and February because of limited surveying, standardized the dataset’s macroinvertebrate taxonomy (e.g., excluded taxa enumerated by just one or two sampling programs, rolled-up genus-level identifications to family level, and updated taxonomic identifications) and rarefied sample counts to approximately 100 individuals per sample event prior to calculating richness and diversity metrics. Family-level macroinvertebrate metrics most sensitive to local habitat and water-quality degradation in each bioregion (regions exhibiting natural differences in undisturbed stream communities) were used to calculate the index. Metric values were scored on a 0–100 scale with a method that identifies reference and degraded sampling events equally. The sampling event’s scores for each metric were then averaged to obtain its index value. The Chessie BIBI was assigned ratings of very poor, poor, fair, good, or excellent, based on thresholds derived from the 10th, 25th, and 50th percentiles of the index scores of each bioregion’s reference samples (Smith et al. 2017). The family-level version of the Chessie BIBI optimizes trade-offs between taxonomic resolution and sample size (i.e., not all monitoring programs identify to genus level). It was selected by Chesapeake Bay Program stakeholders as their preferred stream health indicator and fair, good, or excellent ratings were identified as a preferred outcome for stream restoration efforts (Buchanan et al. 2018). Thus, to align model results with management and restoration goals of a binary category of failing/meeting criteria, we reclassified the 5 categories into 2 broader categories of “degraded” (very poor or poor) and nondegraded (fair, good, or excellent) macroinvertebrate condition. For more information on the dataset, metrics, and Chessie BIBI see Smith et al. (2017) and Maloney et al. (2018).

The rarefaction procedure randomly deletes some taxa from each sample, so we computed BIBI values for each of 100 bootstrapped rarefied subsamples, then considered the median BIBI value as a stable estimate of biological condition in our analyses. In total, 21,266 stream sampling events were available (22 sites had incorrect latitudes or longitudes) with the family-level, bioregion-scale Chessie BIBI, and each was linked to the nearest NHDPlus v2.1 dataset flowline. We merged the Chessie BIBI and HM datasets by the NHDPlus v2.1 unique identifier (COMID) to obtain a predicted estimate of hydrologic alteration for each benthic macroinvertebrate sampling site. We removed sites with incomplete data following the merger (*n* = 7), that had drainages ≥200 km^2^ (*n* = 2007), and when more than one sample event was on a reach (duplicates, *n* = 11,634), we randomly retained one event (*n* = 7618 independent sites). Finally, because some monitoring jurisdictions had a disproportionate density of sampled sites (Fig. S2), we randomly subset these data (*n* = 3799, area = 25,954 km^2^) to align with the spatial density of the remainder of the watershed (*n* = 3819, area = 140,977 km^2^ or 0.027 sites km^–2^), which resulted in a final dataset with 4522 independent sites (1742 in degraded condition and 2780 in nondegraded condition).

### Linking Altered Flow to Biological Condition

To first test for a relationship between flow alteration and macroinvertebrate condition, we generated 2 × 2 contingency tables of flow alteration and Chessie BIBI categories (“degraded,” “nondegraded”), but for different subsets of the dataset as described below. For flow alteration, we created a single binary indicator (“altered” or “unaltered”) for each of the 12 HMs. We did this because our first hypothesis tested overall flow alteration and macroinvertebrate condition and not specific components of the flow regime; this also simplified analysis and interpretation. Each reach was assigned “altered” for each predicted HM if the HM classified it as inflated or diminished, otherwise the reach was assigned “unaltered.” We then summed the number of HMs that were classified as altered for each reach, resulting in a score that ranged from 0 to 12. We interpret this score as the “intensity” of streamflow alteration because it indicates how many components of the flow regime have been modified. We evaluated the hypothesis that biological condition was associated with the intensity of streamflow alteration by testing for a significant association between biological degradation and hydrological alteration using Fisher’s Exact Test for Count Data (fisher.test function, R Core Team 2020) on the contingency table for each numeric value of the intensity score. This test provides an odds ratio (odds in favor for a degraded condition divided by the odds in favor for a nondegraded condition) and we interpreted a significant (*p* < 0.05) odds ratio that was also >1 (considering 95% confidence intervals) to indicate macroinvertebrate communities are more likely to be degraded in hydrologically altered stream segments relative to hydrologically unaltered segments.

We performed the series of Fishers Exact Tests on the entire Chesapeake dataset (*n* = 4522) and also on subsets that represent urban- and agriculture-focused land-use settings. Given the accuracy of NLCD products (Wickham et al. 2017), we operationally defined an urban site as having >5% urban development, <5% of agriculture, and <5% of open water in its upstream watershed, and an agriculture site was defined as having >5% agriculture, <5% urban development, and <5% of open water in upstream watersheds. In addition, to reduce the effects of large dams, each site also needed to have <5 ML/km^−2^ of dam storage in its upstream watershed. Following these selection criteria, we had 300 urban sites and 955 agriculture sites (see Table S2 for land-cover statistical summaries by dataset). We used the results from different land-use settings to provide insight into the relative importance of hydrologic alteration compared to other anthropogenic influences. For example, the odds ratio in the agriculture- or urban-focused watershed dataset may approximate the effects of flow alteration in the presence of either of these two anthropogenic influences.

To more formally test for the relative importance of streamflow alteration and compare it to other anthropogenic influences, we used logistic regression with Chessie BIBI (degraded/nondegraded) as the response. Coefficients from logistic regression also can be reported in the form of odds ratios; in our model, odds ratios for categorical predictors indicate how much more likely a degraded condition is in each category relative to a baseline category, and for continuous predictors, they infer the number of times more likely a degraded condition is given a unit increase in each predictor variable. For predictors, we included the 12-level flow-intensity alteration score, the 15 predictor variables used in the HM random-forest models, bioregion where the Chessie BIBI site resided, and 11 geospatial indicators of natural factors that characterized topography, climate, soil, and lithology (Table S1, Wieczorek et al. 2018). The 12-level flow-alteration score in this analysis was an indicator of flow-alteration intensity because we assumed that sites with more altered HMs are more “intensely” hydrologically altered. We had no a priori reason to select any bioregion as a baseline and thus used Blue Ridge (BR) as the default (alphabetically first) contrast for Bioregion in the model. Selected landscape predictors were identified in previous studies to be important drivers of stream condition (Hill et al. 2017) and the Chessie BIBI (Maloney et al. 2018; Maloney et al. 2020). Exploratory model testing found that incorporating nonlinear effects did not improve the ability to accurately predict Chessie BIBI scores in a validation dataset (Table S3); therefore, we built a single logistic regression model that included only linear effects (glm function in R with a binomial logit link function). This model enabled us to test the additional effects of landscape predictors on whether a degraded macroinvertebrate community is more likely in a hydrologically altered stream. We trained the model with 75% of the data (*n* = 3391) and used the stepAIC function in the MASS R package (Venables and Ripley 2002) to find the best model based on Akaike information criteria. The model was evaluated for fit with Nagelkerke’s pseudo *R*^2^ and Hosmer and Lemeshow goodness-of-fit (GOF) test (hoslem.test in the ResourceSelection R package, Lele et al. 2019) and we validated the model’s accuracy with the independent test dataset (25% of data, *n* = 1131) using AUC, kappa, accuracy, sensitivity (true positive rate), and specificity (true negative rate) with the presence.absence.accuracy function from the PresenceAbsence R package (Freeman and Moisen 2008). Prior to accuracy statistic analyses, the confusion table was optimized based on maximizing kappa with the optimal.thresholds function.

### Paired Gage and Benthic Macroinvertebrate Sites

**Fig. 2:**
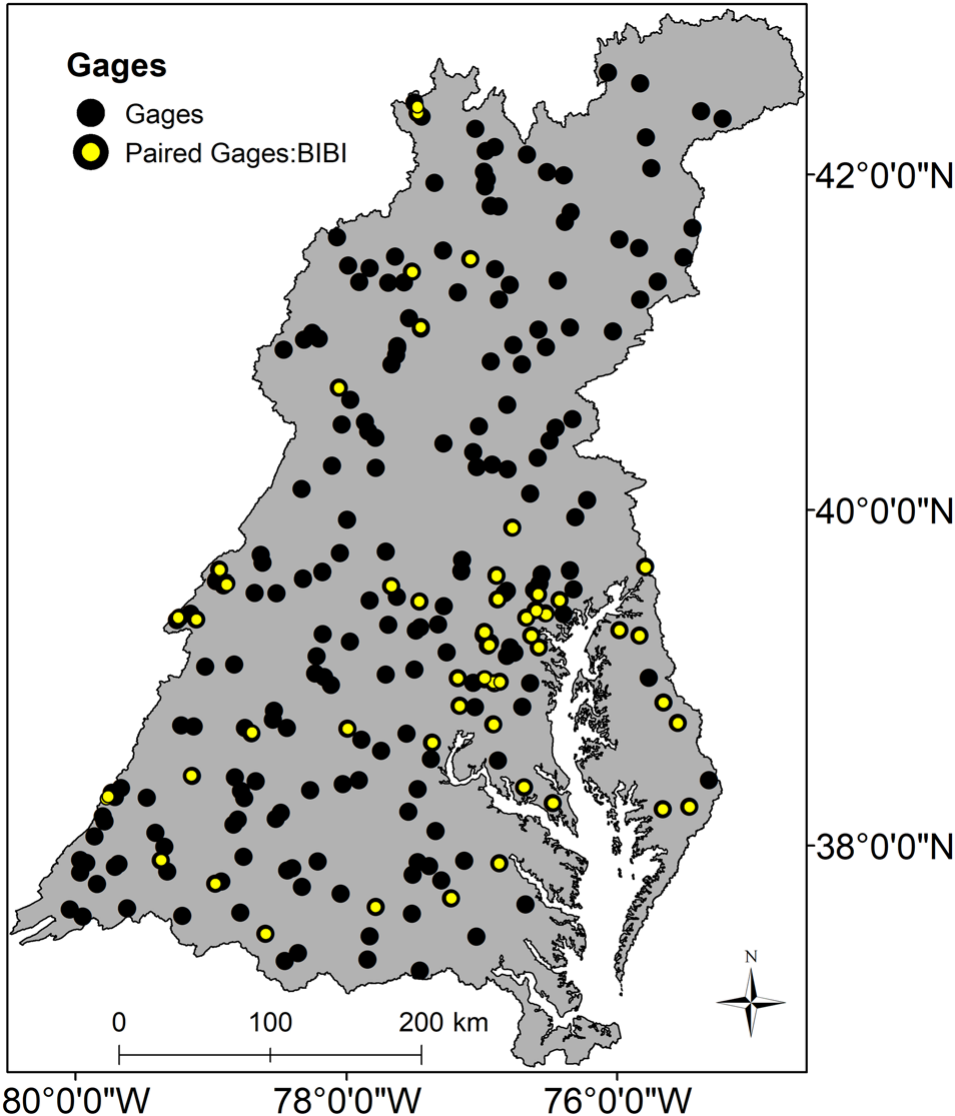
Map showing spatial position of USGS gages from Eng et al. (2019) within the Chesapeake Bay watershed with those paired to a Chessie BIBI and in a small stream (<200-km^2^ upstream drainage) highlighted in yellow

Within the Chesapeake Bay watershed, there were 247 gages with calculated HMs from Eng et al. (2019), of these 50 were in small streams (<200-km^2^ upstream drainage) and were confidently paired with a macroinvertebrate sampling site. Geographic clustering was apparent as 27 of the 50 paired sites were within the state of Maryland (Fig. 2). We used this dataset to examine the likelihood of a degraded stream condition in a flow-altered site for both the observed (Eng et al. 2019) and our modeled estimates of flow-alteration class (altered, unaltered) for each of the 12 HMs using contingency tables (flow-alteration class × stream condition) and Fisher’s Exact Tests for significant odds ratios as specified above. Limited sample size and lack of samples in each bioregion (Table S4), given its importance in previous studies (Maloney et al. 2018, 2020), precluded use of a logistic regression model.

## Results

### HMs and Alteration

For the entire set of gages, random-forest models predicting altered HM were moderately accurate with AUC values ranging from 0.75 (HF_VAR, see Table 1 for HM definitions) to 0.86 (HF_DUR), kappa ranging from 0.37 (HF_VAR) to 0.60 (HF_DUR), and accuracy ranging from 0.60 (HF_VAR) to 0.74 (HF_DUR, Table 3). The model for HF_VAR was the only one to have a kappa <0.40 (fair agreement) and models for HF_VAR and LF_VAR had low sensitivity (<0.40) in predicting inflated conditions, and models for LF_FRE and RISES had low sensitivity in predicting diminished conditions (Table 3). Variable importance values indicated that, except for urban in the HF_MAG, SKEW, and RISES models, urban cover and dam storage were among the most influential predictors for all models (Table 4). Drainage area was an influential predictor of HF_MAG, HF_VAR, SKEW, and RISES. Density of National Pollutant Discharge Elimination System (NPDES) locations (Density NPDES) was an influential predictor of LF_DUR, LF_MAG, LF_VAR, and RISES. Open water cover was an influential predictor of HF_DUR, HF_FRE, HF_MAG, LF_FRE, LF_SEA, and SKEW. In all random-forest models, the three most influential predictors were related to flow alteration in nonlinear ways (Figs. S3–S5). For HF_MAG, the probability of an Indeterminant class initially decreased but then increased with drainage area, and for HF_SEA, the probability of an Indeterminant class was initially flat up to about 40% forest cover and then increased (Fig. S3); for all other relationships in all HMs, the top three important variables showed a strong negative trend followed by a flattening pattern (Figs. S3–S5).

**Table 4:** Variable importance values for each hydrologic metric random-forest model

Landscape predictor	HF_DUR	HF_FRE	HF_MAG	HF_SEA	HF_VAR	LF_DUR	LF_FRE	LF_MAG	LF_SEA	LF_VAR	SKEW	RISES
Tiles	0.0084	0.0104	0.0049	0.0103	0.0054	0.0031	0.0026	0.0044	0.0039	0.0029	0.0068	0.0032
Dam storage	***0.0904***	***0.0523***	***0.0705***	***0.0376***	***0.0755***	***0.0491***	***0.0557***	***0.0666***	***0.0580***	***0.0401***	***0.0585***	***0.0544***
Drainage area	0.0479	0.0259	***0.0386***	0.0320	***0.0383***	0.0134	0.0231	0.0225	0.0081	0.0140	***0.0368***	***0.0353***
Freshwater withdrawal	0.0202	0.0188	0.0109	0.0220	0.0141	0.0131	0.0146	0.0127	0.0147	0.0051	0.0074	0.0070
Canal/ditch/pipeline	0.0116	0.0119	0.0061	0.0042	0.0037	0.0051	0.0026	0.0053	0.0031	0.0024	0.0033	0.0035
Density NPDES locations	0.0278	0.0204	0.0209	0.0280	0.0270	***0.0225***	0.0271	***0.0373***	0.0232	***0.0393***	0.0275	***0.0285***
N application	0.0129	0.0120	0.0104	0.0110	0.0114	0.0059	0.0080	0.0085	0.0102	0.0087	0.0101	0.0053
Pesticide application	0.0065	0.0083	0.0049	0.0068	0.0082	0.0041	0.0054	0.0101	0.0061	0.0038	0.0058	0.0047
Open water	***0.0587***	***0.0512***	***0.0419***	0.0238	0.0208	0.0203	***0.0300***	0.0225	***0.0444***	0.0178	***0.0304***	0.0253
Urban	***0.0607***	***0.0443***	0.0352	***0.0710***	***0.0381***	***0.0521***	***0.0629***	***0.0312***	***0.0509***	***0.0372***	0.0303	0.0221
Barren land	0.0084	0.0118	0.0109	0.0123	0.0115	0.0097	0.0098	0.0126	0.0094	0.0099	0.0131	0.0108
Forest	0.0353	0.0288	0.0228	***0.0376***	0.0141	0.0160	0.0211	0.0249	0.0204	0.0155	0.0164	0.0108
Shrub/scrub	0.0095	0.0106	0.0092	0.0107	0.0102	0.0055	0.0050	0.0113	0.0080	0.0079	0.0081	0.0079
Grassland/herbaceous	0.0125	0.0141	0.0089	0.0152	0.0055	0.0058	0.0094	0.0063	0.0105	0.0039	0.0109	0.0046
Agriculture	0.0140	0.0123	0.0127	0.0114	0.0106	0.0040	0.0056	0.0076	0.0076	0.0054	0.0107	0.0064
Wetland	0.0168	0.0167	0.0065	0.0129	0.0059	0.0066	0.0047	0.0054	0.0062	0.0013	0.0075	0.0069

Bold and italicized indicate the top three variables in each model. Predictor abbreviations defined in Table S1

For the subset of gages in smaller (<200 km^2^) watersheds, HF_MAG, HF_VAR, LF_MAG, LF_VAR, and SKEW had kappa values <0.40 (Table S5 and S6) and models for HF_SEA, LF_DUR, LF_SEA, and LF_VAR had low sensitivity in predicting inflated conditions and models for HF_FRE, HF_VAR, LF_FRE, LF_MAG, and SKEW had low sensitivity of predicting diminished conditions.

Altered flows were prevalent in many reaches in the Northern Piedmont and Southeastern Plains Level III ecoregions (Figs S6–S8). Piedmont, Middle Atlantic Coastal Plain, and the Ridge and Valley Level III ecoregions had concentrated clusters of their areas covered by altered flows, whereas in all other ecoregions, altered flows were more sporadically spread across the landscape.

### Linking Altered Flow to Biological Condition

#### Associations of Altered Streamflow and Macroinvertebrate Condition

**Fig. 3:**
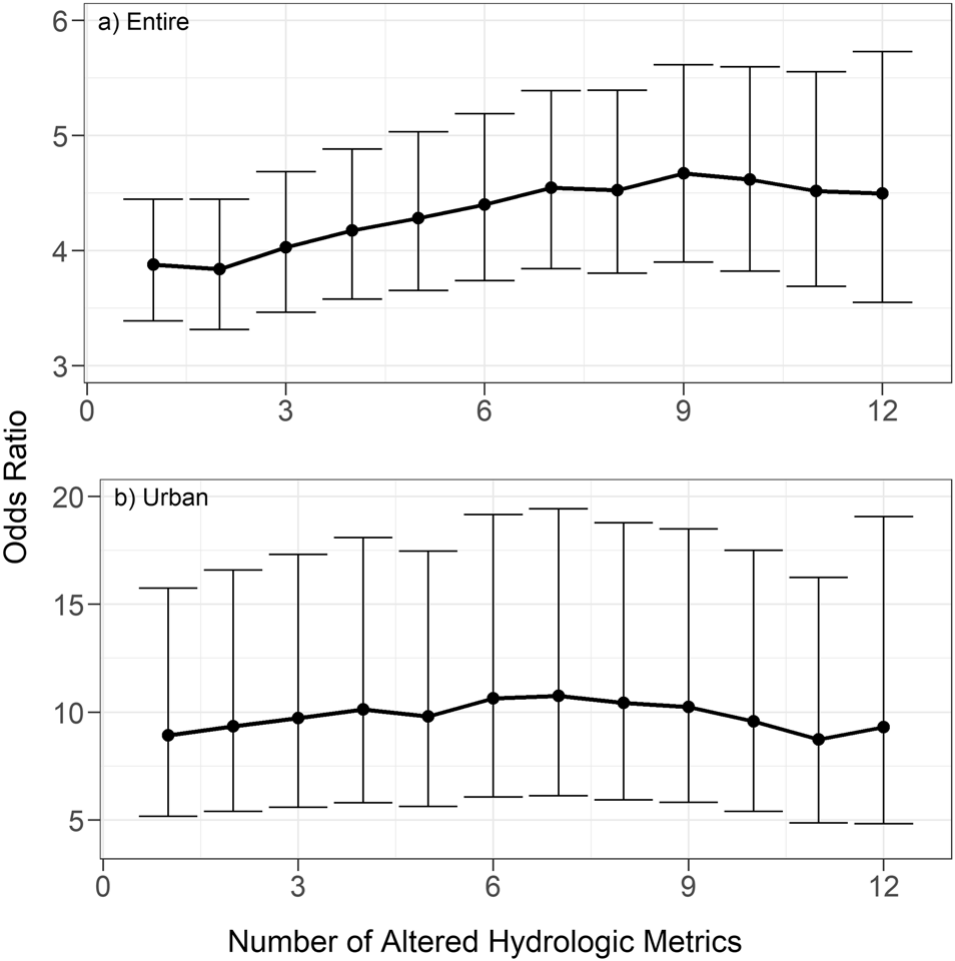
Odds ratio of a degraded macroinvertebrate condition with increasing number of altered hydrologic metrics used to identify an altered flow class (flow-alteration intensity score) for **a** the entire dataset, and **b** subset of data focused on urban development. Subset of data focused on agricultural development not shown because of lack of significant results. Values are presented in Table S7. The odds ratio indicates the increased likelihood that macroinvertebrate communities are degraded in streams with altered flows

For the entire and urban-focused datasets, the odds ratios, or likelihood, of a degraded macroinvertebrate condition increased as the count of flow-altered HMs (indicating the intensity of flow alteration) at a site increased (Fig. 3 and Table S7). No significant odds ratio was detected for the agriculture-focused dataset (Table S7). Across the entire dataset, macroinvertebrate communities were 3.8–4.7 times more likely to be degraded in hydrologically altered sites than in hydrologically unaltered sites (Fig. 3a and Table S7). In the urban-focused dataset, macroinvertebrate communities were 8.7–10.8 times more likely to be degraded in hydrologically altered sites than in hydrologically unaltered sites (Fig. 3b).

#### Influence of Landscape Variables and Altered Flow on Macroinvertebrate Condition

**Fig. 4:**
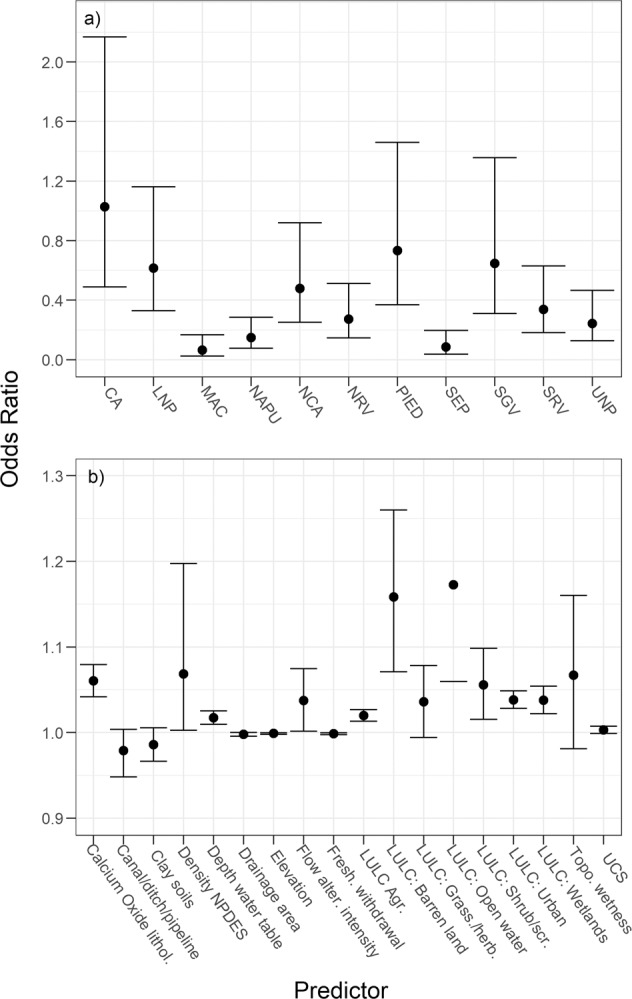
Odds ratios of a degraded macroinvertebrate condition from the logistic regression model using the entire dataset for **a** the categorical predictor bioregion, where an odds ratio indicates how much more likely a degraded condition is in each category relative to the baseline Blue Ridge bioregion, and **b** for the continuous predictors, where odds ratios infer the number of times more likely a degraded condition is given a unit increase in each predictor variable. Error bars are 95% confidence intervals around the estimated odds ratio. Values above one indicate an increased likelihood of a degraded condition. Values are presented in Table S8. Bioregion abbreviations: CA Central Appalachians, LNP Lower-Northern Piedmont, MAC Middle Atlantic Coastal Plain, NAPU Northern Appalachian Plateau and Uplands, NCA North Central Appalachians, NRV Northern Ridge and Valley, PIED Piedmont, SEP Southeastern Plains, SGV Southern Great Valley, SRV Southern Ridge and Valley, and UNP Upper-Northern Piedmont. Predictor abbreviations: lithol. lithology, NPDES National Pollutant Discharge Elimination System, alter. alteration, Fresh. freshwater, LULC land use/Land cover, Agr. agriculture, Grass. grassland, herb. herbaceous, scr. scrub, Topo. topographic, UCS uniaxial compressive strength

The best logistic regression model consisted of 19 predictors (Table S8), had a Nagelkerke *R*^2^ of 0.36, and nonsignificant Hosmer and Lemshow GOF (*p* = 0.73) indicating a good fit of the model (Table S3). The flow-alteration intensity score had a significant coefficient of 0.0367 and an odds ratio of 1.037 (Fig. 4 and Table S8) indicating, controlling for all other predictors, the odds of a degraded macroinvertebrate condition increased 3.7% for every increase in the flow-alteration intensity score. In relation to the BR bioregion, MAC, NAPU, NCA, NRV, SEP, SRV, and UNP (see Fig. 4 for bioregion definitions) all had significant negative coefficients indicating that they are less likely to have a degraded macroinvertebrate condition (odds ratios ranged from 0.06 (MAC) to 0.48 (NCA, Fig. 4 and Table S8). Drainage area, total freshwater withdrawals, and elevation had negative coefficients, whereas open water, barren land, calcium oxide lithology, grassland/herbaceous, urban development, wetlands, agriculture, and depth to the water table had positive coefficients. For a one-unit increase in drainage area, total freshwater withdrawals, and elevation, we expect to see about 0.2%, 0.1%, and 0.1% decrease (respectively) in the likelihood of a site’s stream macroinvertebrate community being degraded, respectively (Table S8). For a one-unit increase in open water, barren land, calcium oxide lithology, grassland/herbaceous, urban development, wetlands, agriculture, and depth to the water table, we expect to see about 17.3%, 15.8%, 6.0%, 5.5%, 3.8%, 3.8%, 2.0%, and 1.7% increase (respectively) in the likelihood of a site’s community being degraded. For the test dataset, the model correctly predicted 267 of the 439 degraded sites (60.8%) and 558 of the 692 (80.6%) not-degraded sites for an overall accuracy of 0.73, kappa = 0.42 (moderate agreement), AUC = 0.77, sensitivity of 0.61, and specificity of 0.81 (Table S3).

### Paired Gage and Benthic Macroinvertebrate Sites

Degraded stream biological conditions were only found to be more likely in one observed HM (SKEW) and one modeled HM (LF_FRE, Table S9).

## Discussion

Accurate predictions of streamflows could dramatically increase the number of reaches where flow-biology relationships can be examined and provide an indication whether altered flow is a possible driver behind degraded conditions. This information is needed to help managers prioritize management practices and more effectively apply restoration efforts across the landscape. This is particularly important in the face of global change, which is predicted to alter the timing and intensity of precipitation in the Chesapeake region (Dupigny-Giroux et al. 2018), and further affect flow regimes. For several thousand ungaged reaches where we paired estimates of predicted flow alteration of 12 HMs that reflect key components of the flow regime with observed benthic macroinvertebrate IBI samples, we found evidence that altered streamflows contribute to biological degradation, and the likelihood of such degradation was higher in urban sites. Further, an increasing intensity of streamflow alteration (defined as an increasing number of attributes of the flow regime) is associated with degraded ecological condition. Paired gage and stream condition sites were both limited and showed a strong spatial pattern, which inhibited detection of consistent flow–ecology relationships. Taken together, our findings suggest that the challenges of assessing flow alteration at regional scales will not only require a reliance on prediction models, but also a thorough evaluation of underlying data (e.g., watershed-size distribution of gages).

### Predicting Hydrologic Alteration

Compared to larger streams, model predictions of the alteration of hydrograph variability, magnitude, and skew were weaker in smaller streams (see Tables 3, S5, and S6). This could be due to several factors that differ between small and large streams, including small streams having relatively fewer gages (Deweber et al. 2014), tighter coupling with hillslope processes, and more temporal and spatial variation (Gomi et al. 2002). Improving all HM predictions for small streams is an avenue for future research, especially for metrics that reflect hydrograph variability, magnitude, and skew. Some avenues of improvement could include better indicators of groundwater influences (Kennen et al. 2014) or enhanced resolution of spatial arrangements of land cover rather than aggregated summaries alone (King et al. 2005; Snyder and Young 2020).

The most highly influential predictor variables of streamflow alteration in our models were consistent with the literature. Previous research highlights the large effect of urban development on the main factors that influence streamflow such as overland flow and infiltration rate (Booth and Jackson 1997; Walsh et al. 2005; Moltz et al. 2018). Water storage reflects the effects of dams and its high importance was expected given the strong effects dams have on flow regimes (Graf 2006; Nilsson et al. 2005; Döll et al. 2009). The influence of dams in the Chesapeake watershed depends on location. For example, the Potomac River, the watershed’s second largest basin, has comparatively few dams and most are run-of-river and minimally alter flow (USACOE et al. 2014). In contrast, flows in the Susquehanna River, the watershed’s largest basin, are more regulated by dams. The importance of drainage area to predictions of altered high flows may be due to the relatively rapid surface runoff rates after storms, which affect high-flow HMs compared to the steadier, slower release rates of groundwater to the surface (baseflow) that affect low-flow HMs. The importance of the density of NPDES locations to the predictions of altered low flows is likely because of effluent discharges during prolonged dry periods when low flows naturally occur. During these periods, effluent releases provide an augmented and relatively constant source of flow, which would inflate low-flow magnitudes but reduce the duration and frequency of low flows—largely because flow magnitudes in these augmented systems rarely fall below the natural thresholds that define low flows (defined in our models as the long-term 10th percentile of natural daily flows).

As has been widely reported (Deweber et al. 2014), the distribution of drainage area was skewed to larger systems in our gage dataset (Fig. S1). However, gages for both the entire dataset and for those gages on small stream reaches (upstream drainage areas <200 km^2^) spanned a wide range of upstream drainage areas across a range of urban development, agriculture, and forest cover (Figs. S9 and S10, respectively). Thus, we had a sufficient dataset of gages to build the predictive models. Yet, we report decreased accuracy in the models in predicting five HMs for small streams, the two indicating magnitude, two indicating variance, and one indicating skew, and note that our models were relatively weak in this area of the hydrograph, possibly a result of the tight linkage of small streams to catchments and a magnified effect of a missing predictor (e.g., groundwater). Documenting the distribution of gages and model performance for our target stream size suggests that gages were not a main factor behind this decreased accuracy. We suggest future studies that model-altered flows in smaller systems should not only document the distribution of gages used in model calibration, but also the accuracy of models for the subset of gages located on smaller reaches.

### Linking Altered Flow to Biological Condition

Large portions of benthic macroinvertebrate taxa are negatively affected by altered flows (Carlisle et al. 2014; Kennen et al. 2014), which results in overall degraded ecological conditions. An earlier version of the Chessie BIBI proved to be sensitive to flow alteration and index scores declined as flow alteration increased (Buchanan et al. 2013). The results from our contingency table analysis generally support this pattern, as results for the entire dataset and urban-focused land-use settings across all levels of flow alteration suggest that degraded macroinvertebrate conditions were more likely in flow-altered sites. When we limited the analysis to watersheds with high levels of urban development, the likelihood of a degraded condition in a flow-altered site increased over twofold compared to those in the entire dataset, suggesting that the effects of urban development on stream biological condition are likely cumulative with other stressors (Walsh et al. 2005; Hughes et al. 2014a, b). This increase in likelihood of degraded conditions in flow-altered sites was not seen in watersheds with varying levels of agriculture, mostly due to limited predicted flow-altered sites in the agriculture-focused dataset. This limited pairing suggests that upstream agriculture may have minimal effect on altering flows beyond natural variability in the Chesapeake Bay watershed—possibly a result of relatively low irrigation in this area (Pervez and Brown 2010)—but more research is needed to confirm this hypothesis.

Benthic macroinvertebrates reflect the cumulative effect of stressors and are driven by a hierarchy of natural landscape filters (Poff 1997). Therefore, the contingency table analyses, although we tried to account for differing land uses, likely missed some important natural and anthropogenic drivers. Our logistic regression model suggests that when using the continuous version of agriculture and when put into context with other landscape variables, the likelihood of a degraded stream condition increased 2.0% for every unit increase in agriculture in upstream watersheds. This, combined with our contingency table analysis where we found no evidence of a flow-alteration effect on stream condition in the agriculture-focused dataset, suggests that other stressors associated with agriculture (e.g., sediment (Noe et al. 2020) and nutrients (Ator et al. 2020)) may be stronger factors affecting macroinvertebrates. Our logistic regression model also supports the importance of other variables as, in addition to the intensity of hydrologic alteration and urban development, we found several natural landscape variables (bioregion, drainage area, elevation, depth to the water table, calcium oxide lithology, % grasslands, and % wetland cover) and additional anthropogenic landscape variables (total freshwater withdrawals, % barren land cover) significant. The decreased likelihood of a degraded macroinvertebrate condition with increasing elevation and increasing likelihood of a degraded macroinvertebrate condition with open water, barren land, and urban development, were expected and are documented in other studies (Davies et al. 2010; Hill et al. 2017; Maloney et al. 2018). Elevation may be serving as a proxy for slope with shallower-sloped lands, which are more prevalent at lower elevations in the Chesapeake watershed and support a larger human population and more degraded benthic communities (USACOE et al. 2014). Spatial patterns could be a driving factor behind the unexpected results with depth to the water table, calcium oxide lithology, water withdrawal, and percent wetland cover but for different reasons. For example, depth to the water table and wetland cover both show strong regional patterns with shallower depth to water tables (Fig. S11A) and higher percentage of wetlands (Fig. S11B) in the Coastal Plains bioregion, which could be a factor behind the increased likelihood of a degraded condition. The Coastal Plains bioregion had few reference sites within the Chesapeake watershed, which may have affected Chessie BIBI calibration and thus the predictive ability of our model. Total freshwater withdrawals had a spatial clustering pattern (Fig. S11C), which was likely a result of the county-level availability of these data. Calcium oxide lithology showed some spatial patterns (Fig. S11D), reflective of catchments with karst geology (Fig. S12). Aquatic habitats differ in karst landscapes in contribution of surface and groundwater, geomorphology, and benthic macroinvertebrate communities (Reid et al. 2012), but Smith et al. (2017) found little difference in metrics used in the Chessie BIBI between reference karst and non-karst sites. Anthropogenic stressors may have a disproportionate effect on benthic macroinvertebrates in karst-dominated streams, but further study is needed to support this hypothesis.

Here, we tested the relationship between altered flows and stream conditions using a measure of alteration intensity—defined as the breadth of alteration across numerous components of the flow regime. To our knowledge, this aspect of streamflow alteration has not been rigorously examined. Our analyses support the notion that streams with a wider range of unnatural conditions are more likely to exhibit degraded benthic communities. This finding coupled with previous studies implies there are at least three dimensions of streamflow alteration that should be considered to understand ecological consequences. First, how do individual attributes of the flow regime that are altered affect associated communities. For example, the frequency of low-flow spells may negatively affect macroinvertebrate species with a good crawling trait (Patrick and Yuan 2017). Second, what is the severity with which each flow attribute has departed from natural conditions and its effects on communities, as demonstrated in Carlisle et al. (2014, 2019). Third, as found here, what is the breadth of flow-altered attributes that influence ecological outcomes. It stands to reason that if an assemblage of species collectively has requirements for a broad range of flow conditions, streams where more flow attributes have been altered are likely to have more extreme ecological degradation.

There are thousands of potential HMs and our limitation to the 12 HMs available from Eng et al. (2019) may miss an important component of the hydrograph. However, these 12 represent the five important components of the flow regime (duration, frequency, magnitude, timing, and rate of change, Poff et al. 1997) and previous studies have found that these 12 are strongly related to benthic macroinvertebrates and stream biological condition (Buchanan et al. 2013; Carlisle et al. 2017). Therefore, our results should be a robust reflection of currently altered flow effects on small stream biological conditions in the Chesapeake Bay watershed. However, in addition to improving the predictive ability of HMs used in this study, our analyses may be improved by future research that focuses on examining additional HMs from a functional flow approach (Yarnell et al. 2020), examines trait-based metrics (Monk et al. 2018), or uses hierarchical approaches (Webb et al. 2018).

**Fig. 5:**
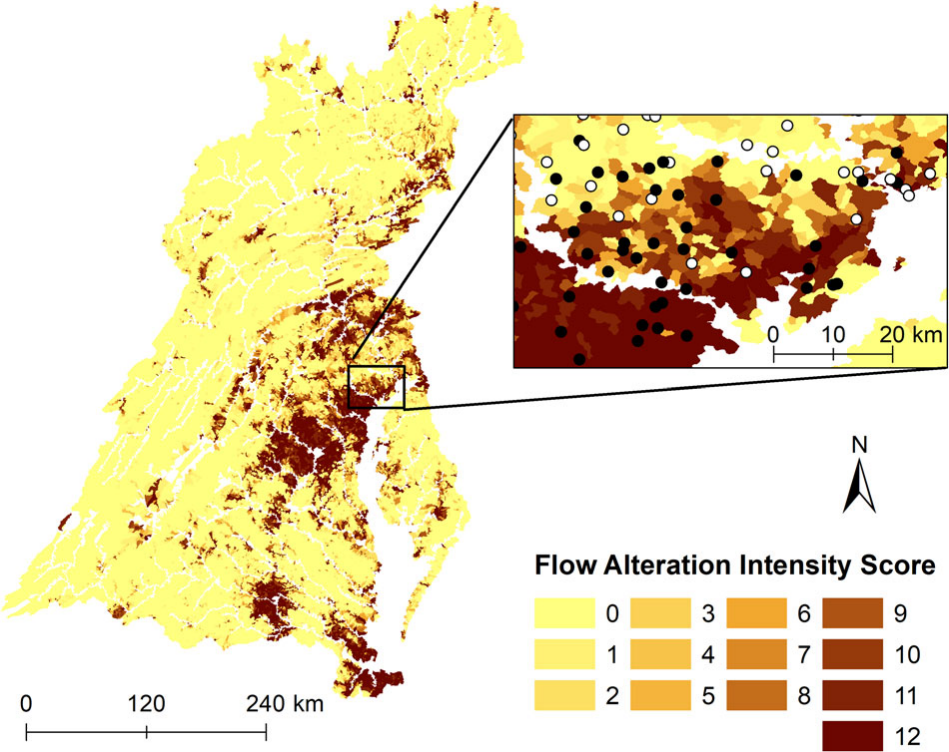
Map of Chesapeake Bay watershed showing the flow-altered intensity score for all small streams (<200 km^2^ in upstream drainage) and a focus area with stream condition overlain

Our single-flow-alteration intensity index when coupled with a measure of stream condition can identify reaches where altered flow is and is not a potential major stressor driving stream degradation. For example, reaches with degraded conditions but not identified with altered flow suggest that other stressors are likely driving degraded conditions (Fig. 5). This could facilitate managers in not only identifying potential restoration sites, but could also assist in identifying the most effective restoration practices (i.e., if no flow alteration is evident, do not prescribe a management practice aimed at this stressor). If flow alteration is the stressor of interest, then areas with a lower flow-alteration intensity index score (lighter colors in Fig. 5) may be more responsive to management practices because of fewer aspects of the hydrograph requiring remediating. We acknowledge that flow is one of many possible stressors and the most effective use of our results will be when they are put into context with other leading stressors for the Chesapeake Bay watershed (e.g., sediment (Noe et al. 2020) and nutrients (Ator et al. 2020)).

Our approach has several limitations. First, as is often the case, we had too few paired gaged and benthic macroinvertebrate sites to perform any rigorous analysis of the altered flow—biological endpoint relationship with observed flow data. As a result, we had to use modeled HMs rather than observed HMs to define alteration, which incorporates model error. Second, our approach produced a reasonably accurate prediction of the breadth of flow alteration at the reach scale for the entire watershed, but it did not produce information on individual components of the hydrograph or stressor-response relationships. Therefore, our results cannot be used to establish management targets. Our goal, however, was to predict the likelihood that streamflow alteration is a contributor to biological degradation, which can aid in the prioritization of restoration management practices. Third, combining flow alteration of each HM into three categories simplifies interpretation, but removes the ability to examine the intensity of change for any single HMs. The focus of this study was not the effect of individual HMs but rather overall hydrologic alteration; however, this simplification may result in undetected relationships between altered flow and stream condition. Fourth, we note that the same 15 anthropogenic landscape variables were used in our logistic regression model that also were used in the random-forest models to predict flow-alteration class. Similar approaches have been previously applied to enable incorporation of a predictor of interest that has no measured data (e.g., Vander Laan and Hawkins 2014); however, doing so has the potential to introduce circularity that may obfuscate inferences. We are unaware of any approach that completely overcomes this problem given our data limitations. We limited the possible influence of circularity by only using a composite index of flow alteration rather than the individual predicted classes for each HM and by incorporating 11 natural landscape variables as well as the bioregion of the stream survey point. However, these approaches may not have completely removed the possibility of circularity and we caution the overinterpretation of logistic regression model output. Fifth, the coarse taxonomic resolution and low subsample count used in the Chessie BIBI could have reduced the sensitivity of the BIBI to flow alteration. Smith et al. (2017) report no large difference in classification efficiency between family and genus-level-derived IBIs, so taxonomic resolution likely did not affect the results. But no such analysis has been conducted for low subsample count, so we cannot rule out a possible effect on sensitivity. Finally, we used a single year for land cover (2011), but biological data from a 23-year period. We acknowledge that land-cover variables change, albeit slowly, over time, which could have added noise to our analyses. We chose 2011 because it was near the end of the biological sampling period and would best reflect land cover that occurs over the span of the biological data. Incorporating a temporal aspect to land use may improve understanding of ecological flow relationships, particularly when long-term biological data are used, but this was beyond the scope of our study.

## Conclusion

Limited paired biological and long-term flow data have often inhibited studies that have directly tested the altered flow-biology relationship. This is particularly true for smaller streams with fewer gaged sites than larger systems. To overcome this limitation, we leveraged a large biological dataset that we paired with modeled estimates of hydrologic alteration. We found that a degraded macroinvertebrate condition was more likely in flow-altered sites and this likelihood increased with increasing flow-alteration intensity even after accounting for other strong landscape drivers. These findings add to our understating of how and where altered flows may be affecting stream conditions in the Chesapeake Bay watershed. A similar approach can be used in other areas with such data limitations.

## Supplementary information

Supplementary Material Files

Supplementary Material TableS1
