# Supplementary material for: Linking Altered Flow Regimes to Biological Condition: an Example Using Benthic Macroinvertebrates in Small Streams of the Chesapeake Bay Watershed
Source: Environ Manage. 2021 Mar 12;67(6):1171–85. doi: 10.1007/s00267-021-01450-5 (PMC8106597; doi:10.1007/s00267-021-01450-5)
Supplement: Supplementary file 1 — Supplementary Material Files [file 267_2021_1450_MOESM1_ESM.docx]

**Supplementary Material for Linking altered flow regimes to biological condition: an example using benthic macroinvertebrates in small streams of the Chesapeake Bay watershed**

Kelly O. Maloney ^1^*

Daren M. Carlisle^2^

Claire Buchanan^3^

Jennifer L. Rapp^4^

Samuel H. Austin^4^

Matthew J. Cashman^5^

John A. Young^1^

^1^U.S. Geological Survey, Leetown Science Center, Kearneysville West Virginia, 25430 USA; ORCID: 0000-0003-2304-0745; 0000-0002-4500-3673

^2^U.S. Geological Survey, Lawrence, Kansas 66049 USA; ORCID: 0000-0002-7367-348X

^3^Interstate Commission on the Potomac River Basin (ICPRB), 30 West Gude Drive, Suite 450, Rockville, Maryland 20850 USA; ORCID: 0000-0001-5627-448X

^4^U.S. Geological Survey, Virginia and West Virginia Water Science Center, Richmond, Virginia 23228.  ORCID: 0000-0003-2253-9886, 0000-0001-5626-023X

^5^U.S. Geological Survey, Maryland-Delaware-District of Columbia Water Science Center, Baltimore, MD, USA; ORCID: 0000-0002-6635-4309

**Supplemental Materials**

Table S1. Summary statistics and description for predictors used in the HM (n =1,235) and Chessie BIBI (training n = 3,391) models and for all reaches in the Chesapeake Bay watershed (n = 82,234). Predictor values from Wieczorek et al. (2018), Bioregion was also included in the Chessie BIBI logistic regression models but is not included in the table. Min. = minimum, Max = maximum, PRISM = Parameter-elevation Regressions on Independent Slopes Model, NPDES = National Pollutant Discharge Elimination System, NHD = National Hydrography Dataset, NLCD = National Land Cover Database.

See separate csv file.

Table S2. Summary statistics for urban and agriculture land cover (2011 National Land Cover Database) by data set. Min = minimum, Max = maximum, Perc. = percentile.

|  |  | Urban Cover | | | | | |  | Agriculture Cover | | | | | |
| --- | --- | --- | --- | --- | --- | --- | --- | --- | --- | --- | --- | --- | --- | --- |
| Data set | n | Mean | Median | Min | Max | 10th Perc. | 90th Perc. |  | Mean | Median | Min | Max | 10th Perc. | 90th Perc. |
| Entire | 4522 | 10.9 | 5.2 | 0.0 | 98.7 | 2.1 | 25.1 |  | 21.6 | 15.5 | 0.0 | 91.3 | 0.3 | 53.9 |
| Agriculture | 955 | 3.6 | 3.8 | 0.0 | 5.0 | 2.2 | 4.8 |  | 22.9 | 17.0 | 5.0 | 91.3 | 7.0 | 49.8 |
| Urban | 300 | 37.0 | 20.0 | 5.0 | 93.7 | 5.4 | 83.1 |  | 1.6 | 1.2 | 0.0 | 5.0 | 0.0 | 4.0 |

Table S3. Results of exploratory non-linear only logistic regression model analyses using the entire data set (n = 3,391 for training and 1,131 as test data set). Reduced models using stepAIC function in R package MASS. Confusion matrices and accuracy statistics for the test data set.

|  |  | Linear Model | |  | Non-linear Model | |
| --- | --- | --- | --- | --- | --- | --- |
| **Training data set accuracy** |  |  |  |  |  |  |
| Nagelkerke's pseudo R^2^* |  | 0.36 | |  | 0.35 | |
| Hosmer and Lemeshow goodness of fit (GOF) test p-value |  | 0.73 | |  | 0.05 | |
|  |  |  |  |  |  |  |
| **Test data set accuracy** |  |  |  |  |  |  |
| Confusion Matrices |  | Observed | |  | Observed | |
|  |  | Degraded | Not Degraded |  | Degraded | Not Degraded |
| Degraded |  | 267 | 134 |  | 260 | 128 |
| Not Degraded |  | 172 | 558 |  | 179 | 564 |
|  |  |  |  |  |  |  |
| Model Fit and Accuracy Statistics |  |  |  |  |  |  |
| Optimized Threshold |  | 0.45 | |  | 0.45 | |
| Accuracy |  | 0.73 | |  | 0.73 | |
| Kappa |  | 0.42 | |  | 0.42 | |
| AUC |  | 0.77 | |  | 0.77 | |
| Sensitivity |  | 0.61 | |  | 0.59 | |
| Specificity |  | 0.81 | |  | 0.82 | |

Table S4. Number of paired gage and Chessie BIBI sites in each bioregion by flow alteration intensity score estimated using observed hydrologic metrics and modeled hydrologic metrics (in parentheses). For flow alteration intensity, scores of 2, 3, 4, and 6 are not displayed because no sites fell in these levels.

|  | Flow Alteration Intensity | | | | | | | | |
| --- | --- | --- | --- | --- | --- | --- | --- | --- | --- |
| Bioregion | 0 | 1 | 5 | 7 | 8 | 9 | 10 | 11 | 12 |
| Blue Ridge | 0 (0) | 0 (0) | 0 (0) | 0 (0) | 0 (0) | 0 (0) | 0 (0) | 0 (0) | 0 (0) |
| Central Appalachians | 1 (2) | 0 (0) | 0 (0) | 1 (0) | 0 (0) | 0 (0) | 1 (1) | 0 (0) | 0 (0) |
| Lower-Northern Piedmont | 1 (1) | 0 (1) | 0 (0) | 0 (0) | 1 (1) | 1 (0) | 1 (0) | 0 (0) | 0 (1) |
| Middle Atlantic Coastal Plain | 4 (5) | 0 (1) | 0 (0) | 0 (0) | 0 (0) | 0 (0) | 2 (0) | 0 (0) | 0 (0) |
| Northern Appalachian Plateau and Uplands | 0 (0) | 0 (0) | 0 (1) | 0 (0) | 0 (0) | 0 (0) | 0 (0) | 2 (1) | 0 (0) |
| North Central Appalachians | 2 (2) | 0 (0) | 0 (0) | 0 (0) | 0 (0) | 0 (0) | 0 (0) | 0 (0) | 0 (0) |
| Northern Ridge and Valley | 2 (2) | 0 (0) | 0 (0) | 0 (0) | 0 (0) | 0 (0) | 0 (0) | 0 (0) | 0 (0) |
| Piedmont | 3 (3) | 0 (0) | 0 (0) | 1 (1) | 0 (0) | 0 (0) | 1 (1) | 0 (0) | 0 (0) |
| Southeastern Plains | 2 (2) | 0 (0) | 0 (0) | 0 (0) | 0 (0) | 0 (0) | 2 (2) | 5 (4) | 2 (3) |
| Southern Great Valley | 0 (0) | 0 (0) | 0 (0) | 0 (1) | 0 (0) | 0 (0) | 1 (1) | 1 (1) | 1 (0) |
| Southern Ridge and Valley | 2 (2) | 0 (0) | 0 (0) | 0 (0) | 0 (0) | 0 (0) | 0 (0) | 1 (1) | 0 (0) |
| Upper-Northern Piedmont | 2 (2) | 0 (0) | 0 (0) | 0 (0) | 0 (0) | 1 (1) | 3 (1) | 3 (4) | 0 (1) |

Table S5. Accuracy statistics for each hydrologic metric for subset of gages with upstream drainage areas <200 km^2^. OOB = out of bag error rate from models, AUC = area under the receiver operation curve. Bold and italicized indicate a Kappa < 0.41 or a Sensitivity or Specificity score < 0.40.

|  |  |  |  | Sensitivity | | |  | Specificity | | |
| --- | --- | --- | --- | --- | --- | --- | --- | --- | --- | --- |
| Metric | AUC | Accuracy | Kappa | Diminished | Indeterminant | Inflated |  | Diminished | Indeterminant | Inflated |
| HF_DUR | 0.87 | 0.77 | 0.62 | 0.81 | 0.89 | 0.45 |  | 0.87 | 0.78 | 0.97 |
| HF_FRE | 0.80 | 0.65 | 0.44 | ***0.37*** | 0.69 | 0.76 |  | 0.91 | 0.68 | 0.85 |
| HF_MAG | 0.76 | 0.58 | ***0.36*** | 0.42 | 0.73 | 0.56 |  | 0.92 | 0.74 | 0.73 |
| HF_SEA | 0.78 | 0.69 | 0.47 | 0.81 | 0.80 | ***0.07*** |  | 0.77 | 0.72 | 0.99 |
| HF_VAR | 0.74 | 0.56 | ***0.32*** | ***0.38*** | 0.75 | 0.47 |  | 0.95 | 0.63 | 0.75 |
| LF_DUR | 0.85 | 0.76 | 0.59 | 0.78 | 0.86 | ***0.27*** |  | 0.85 | 0.84 | 0.93 |
| LF_FRE | 0.79 | 0.71 | 0.54 | ***0.31*** | 0.86 | 0.73 |  | 0.90 | 0.77 | 0.89 |
| LF_MAG | 0.76 | 0.56 | ***0.33*** | ***0.34*** | 0.86 | 0.42 |  | 0.84 | 0.66 | 0.83 |
| LF_SEA | 0.76 | 0.67 | 0.46 | 0.70 | 0.85 | ***0.19*** |  | 0.76 | 0.76 | 0.94 |
| LF_VAR | 0.74 | 0.63 | ***0.40*** | 0.57 | 0.80 | ***0.25*** |  | 0.83 | 0.74 | 0.85 |
| SKEW | 0.75 | 0.57 | ***0.32*** | ***0.25*** | 0.77 | 0.55 |  | 0.95 | 0.65 | 0.72 |
| RISES | 0.79 | 0.66 | 0.47 | 0.53 | 0.87 | 0.53 |  | 0.87 | 0.73 | 0.89 |

Table S6. Confusion matrices for observed and predicted flow alteration for each of the 12 hydrologic metrics for all sites and subset of sites with upstream drainages <200 km^2^. Bold and italicized highlight rows where misclassified predictions were higher than correctly classified.

|  |  | Predicted Flow Alteration | | | | | | |
| --- | --- | --- | --- | --- | --- | --- | --- | --- |
| Hydrologic Metric | Observed Flow Alteration |  | All sites |  |  | Only sites <200 km2 upstream drainage | | |
|  |  | Diminished | Indeterminant | Inflated |  | Diminished | Indeterminant | Inflated |
| HF_DUR | Diminished | 53 | 7 | 16 |  | 35 | 3 | 4 |
|  | Indeterminant | 15 | 72 | 17 |  | 7 | 31 | 7 |
|  | Inflated | 10 | 15 | 104 |  | 1 | 1 | 9 |
| HF_FRE | Diminished | 98 | 20 | 18 |  | ***7*** | ***7*** | ***0*** |
|  | Indeterminant | 19 | 70 | 21 |  | 9 | 29 | 9 |
|  | Inflated | 9 | 9 | 45 |  | 3 | 6 | 28 |
| HF_MAG | Diminished | 123 | 24 | 9 |  | 15 | 3 | 2 |
|  | Indeterminant | 21 | 71 | 9 |  | 8 | 32 | 6 |
|  | Inflated | ***21*** | ***14*** | ***17*** |  | ***13*** | ***9*** | ***10*** |
| HF_SEA | Diminished | 66 | 23 | 15 |  | 34 | 8 | 5 |
|  | Indeterminant | 21 | 78 | 16 |  | 7 | 33 | 9 |
|  | Inflated | 11 | 25 | 54 |  | ***1*** | ***0*** | ***1*** |
| HF_VAR | Diminished | 96 | 26 | 16 |  | 14 | 3 | 0 |
|  | Indeterminant | 20 | 70 | 15 |  | 11 | 33 | 9 |
|  | Inflated | ***30*** | ***16*** | ***20*** |  | ***12*** | ***8*** | ***8*** |
| LF_DUR | Diminished | 112 | 15 | 9 |  | 39 | 5 | 3 |
|  | Indeterminant | 22 | 73 | 9 |  | 5 | 38 | 5 |
|  | Inflated | ***31*** | ***14*** | ***23*** |  | ***6*** | ***1*** | ***3*** |
| LF_FRE | Diminished | ***24*** | ***17*** | ***21*** |  | ***5*** | ***3*** | ***5*** |
|  | Indeterminant | 14 | 68 | 14 |  | 7 | 32 | 7 |
|  | Inflated | 22 | 14 | 115 |  | 4 | 2 | 33 |
| LF_MAG | Diminished | 36 | 8 | 23 |  | ***10*** | ***2*** | ***9*** |
|  | Indeterminant | 16 | 72 | 17 |  | 11 | 31 | 10 |
|  | Inflated | 21 | 18 | 98 |  | 8 | 3 | 14 |
| LF_SEA | Diminished | 105 | 12 | 28 |  | 30 | 5 | 8 |
|  | Indeterminant | 15 | 70 | 18 |  | 9 | 33 | 5 |
|  | Inflated | 11 | 18 | 32 |  | ***4*** | ***1*** | ***3*** |
| LF_VAR | Diminished | 122 | 18 | 15 |  | 24 | 6 | 5 |
|  | Indeterminant | 17 | 76 | 16 |  | 8 | 40 | 7 |
|  | Inflated | ***19*** | ***12*** | ***14*** |  | ***10*** | ***4*** | ***4*** |
| SKEW | Diminished | 116 | 25 | 6 |  | 6 | 2 | 2 |
|  | Indeterminant | 24 | 72 | 15 |  | 7 | 33 | 12 |
|  | Inflated | ***18*** | ***12*** | ***21*** |  | ***11*** | ***8*** | ***17*** |
| RISES | Diminished | ***8*** | ***9*** | ***16*** |  | ***8*** | ***1*** | ***10*** |
|  | Indeterminant | 9 | 71 | 28 |  | 5 | 33 | 11 |
|  | Inflated | 12 | 22 | 134 |  | 2 | 4 | 24 |

Table S7. Results of Fisher’s Exact Test for each data set for each level of the flow alteration intensity score depicting the odds of a degraded macroinvertebrate condition in a flow-altered site. Not Degr. = Not Degraded, Degr. = Degraded, NA = Fisher’s Exact test not run due to 0 flow-altered sites.

|  |  | Entire | | | | |  | Urban | | | | |  | Agriculture | | | | |
| --- | --- | --- | --- | --- | --- | --- | --- | --- | --- | --- | --- | --- | --- | --- | --- | --- | --- | --- |
| HM Cutoff |  | Stream Condition | |  | Fisher's Exact Test | |  | Stream Condition | |  | Fisher's Exact Test | |  | Stream Condition | |  | Fisher's Exact Test | |
|  | Flow | Not Degr. | Degr. |  | p-value | Odds Ratio |  | Not Degr. | Degr. |  | p-value | Odds Ratio |  | Not Degr. | Degr. |  | p-value | Odds Ratio |
| 1 | Not Altered | 2253 | 913 |  | < 2.2e-16 | 3.9 |  | 111 | 39 |  | < 2.2e-16 | 8.9 |  | 640 | 299 |  | 1 | 1.0 |
|  | Altered | 527 | 829 |  |  | (3.4 - 4.4) |  | 36 | 114 |  |  | (5.2 - 15.7) |  | 11 | 5 |  |  | (0.3 - 3.1) |
|  |  |  |  |  |  |  |  |  |  |  |  |  |  |  |  |  |  |  |
| 2 | Not Altered | 2390 | 1071 |  | < 2.2e-16 | 3.8 |  | 114 | 41 |  | < 2.2e-16 | 9.4 |  | 648 | 302 |  | 0.6559 | 1.4 |
|  | Altered | 390 | 671 |  |  | (3.3 - 4.4) |  | 33 | 112 |  |  | (5.4 - 16.6) |  | 3 | 2 |  |  | (0.1 - 12.5) |
|  |  |  |  |  |  |  |  |  |  |  |  |  |  |  |  |  |  |  |
| 3 | Not Altered | 2429 | 1101 |  | < 2.2e-16 | 4.0 |  | 115 | 41 |  | < 2.2e-16 | 9.7 |  | 648 | 303 |  | 1 | 0.7 |
|  | Altered | 351 | 641 |  |  | (3.5-4.7) |  | 32 | 112 |  |  | (5.6 - 17.3) |  | 3 | 1 |  |  | (0.01 - 8.9) |
|  |  |  |  |  |  |  |  |  |  |  |  |  |  |  |  |  |  |  |
| 4 | Not Altered | 2461 | 1130 |  | < 2.2e-16 | 4.2 |  | 116 | 41 |  | < 2.2e-16 | 10.1 |  | 650 | 303 |  | 0.5355 | 2.1 |
|  | Altered | 319 | 612 |  |  | (3.6- 4.9) |  | 31 | 112 |  |  | (5.8 - 18.1) |  | 1 | 1 |  |  | (0.03 - 168.4) |
|  |  |  |  |  |  |  |  |  |  |  |  |  |  |  |  |  |  |  |
| 5 | Not Altered | 2488 | 1159 |  | < 2.2e-16 | 4.3 |  | 116 | 42 |  | < 2.2e-16 | 9.8 |  | 650 | 304 |  | 1 | 0.0 |
|  | Altered | 292 | 583 |  |  | (3.7 - 5.0) |  | 31 | 111 |  |  | (5.6 - 17.5) |  | 1 | 0 |  |  | (0.0 - 83.4) |
|  |  |  |  |  |  |  |  |  |  |  |  |  |  |  |  |  |  |  |
| 6 | Not Altered | 2508 | 1179 |  | < 2.2e-16 | 4.4 |  | 118 | 42 |  | < 2.2e-16 | 10.6 |  | 651 | 304 |  | 1 | NA |
|  | Altered | 272 | 563 |  |  | (3.7 - 5.2) |  | 29 | 111 |  |  | (6.1 - 19.2) |  | 0 | 0 |  |  |  |
|  |  |  |  |  |  |  |  |  |  |  |  |  |  |  |  |  |  |  |
| 7 | Not Altered | 2532 | 1205 |  | < 2.2e-16 | 4.5 |  | 119 | 43 |  | < 2.2e-16 | 10.8 |  | 651 | 304 |  | 1 | NA |
|  | Altered | 248 | 537 |  |  | (3.8 - 5.4) |  | 28 | 110 |  |  | (6.1 - 19.4) |  | 0 | 0 |  |  |  |
|  |  |  |  |  |  |  |  |  |  |  |  |  |  |  |  |  |  |  |
| 8 | Not Altered | 2553 | 1242 |  | < 2.2e-16 | 4.5 |  | 119 | 44 |  | < 2.2e-16 | 10.4 |  | 651 | 304 |  | 1 | NA |
|  | Altered | 227 | 500 |  |  | (3.8 - 5.4) |  | 28 | 109 |  |  | (5.9 - 18.8) |  | 0 | 0 |  |  |  |
|  |  |  |  |  |  |  |  |  |  |  |  |  |  |  |  |  |  |  |
| 9 | Not Altered | 2578 | 1275 |  | < 2.2e-16 | 4.7 |  | 120 | 46 |  | < 2.2e-16 | 10.2 |  | 651 | 304 |  | 1 | NA |
|  | Altered | 202 | 467 |  |  | (3.9 - 5.6) |  | 27 | 107 |  |  | (5.8 -18.5) |  | 0 | 0 |  |  |  |
|  |  |  |  |  |  |  |  |  |  |  |  |  |  |  |  |  |  |  |
| 10 | Not Altered | 2599 | 1318 |  | < 2.2e-16 | 4.6 |  | 123 | 53 |  | < 2.2e-16 | 9.6 |  | 651 | 304 |  | 1 | NA |
|  | Altered | 181 | 424 |  |  | (3.8 -5.6) |  | 24 | 100 |  |  | (5.4 - 17.5) |  | 0 | 0 |  |  |  |
|  |  |  |  |  |  |  |  |  |  |  |  |  |  |  |  |  |  |  |
| 11 | Not Altered | 2626 | 1377 |  | < 2.2e-16 | 4.5 |  | 126 | 62 |  | 2.36E-16 | 8.7 |  | 651 | 304 |  | 1 | NA |
|  | Altered | 154 | 365 |  |  | (3.7 - 5.6) |  | 21 | 91 |  |  | (4.9 - 16.2) |  | 0 | 0 |  |  |  |
|  |  |  |  |  |  |  |  |  |  |  |  |  |  |  |  |  |  |  |
| 12 | Not Altered | 2672 | 1474 |  | < 2.2e-16 | 4.5 |  | 133 | 77 |  | 7.04E-15 | 9.3 |  | 651 | 304 |  | 1 | NA |
|  | Altered | 108 | 268 |  |  | (3.5 - 5.7) |  | 14 | 76 |  |  | (4.8 - 19.1) |  | 0 | 0 |  |  |  |

Table S8. Results of logistic regression model using the entire data set (training data set, n = 3,391), Bold and italicized highlight predictors significant at p = 0.05. Blue Ridge is the default contrast for Bioregion in the model. Dashed line (-----) indicates Odds Ratio not calculated.

| Variable | Estimate | Std. Error | Lower | Upper | z value | Estimate | Lower CI | Upper CI | p-value |
| --- | --- | --- | --- | --- | --- | --- | --- | --- | --- |
| Intercept | -0.6574 | 0.6884 | -2.0088 | 0.6910 | -0.955 | **-----** | **-----** | **-----** | 0.339594 |
| Flow alteration intensity | **0.0367** | **0.0179** | **0.0016** | **0.0718** | **2.052** | **1.037** | **1.002** | **1.074** | **0.040164** |
| Bioregion: Central Appalachians | 0.0259 | 0.3794 | -0.7148 | 0.7744 | 0.068 | 1.026 | 0.489 | 2.169 | 0.94551 |
| Bioregion: Lower-Northern Piedmont | -0.4841 | 0.3209 | -1.1110 | 0.1492 | -1.508 | 0.616 | 0.329 | 1.161 | 0.131494 |
| Bioregion: Middle Atlantic Coastal Plain | **-2.7433** | **0.4885** | **-3.7040** | **-1.7876** | **-5.616** | **0.064** | **0.025** | **0.167** | **1.95E-08** |
| Bioregion: Northern Appalachian Plateau and Uplands | **-1.9121** | **0.3356** | **-2.5681** | **-1.2507** | **-5.698** | **0.148** | **0.077** | **0.286** | **1.21E-08** |
| Bioregion: North Central Appalachians | **-0.7374** | **0.3308** | **-1.3825** | **-0.0837** | **-2.229** | **0.478** | **0.251** | **0.920** | **0.025802** |
| Bioregion: Northern Ridge and Valley | **-1.2998** | **0.3195** | **-1.9237** | **-0.6689** | **-4.068** | **0.273** | **0.146** | **0.512** | **4.74E-05** |
| Bioregion: Piedmont | -0.3099 | 0.3498 | -0.9945 | 0.3788 | -0.886 | 0.733 | 0.370 | 1.461 | 0.375655 |
| Bioregion: Southeastern Plains | **-2.4589** | **0.4274** | **-3.2985** | **-1.6211** | **-5.753** | **0.086** | **0.037** | **0.198** | **8.75E-09** |
| Bioregion: Southern Great Valley | -0.4345 | 0.3756 | -1.1688 | 0.3058 | -1.157 | 0.648 | 0.311 | 1.358 | 0.247369 |
| Bioregion: Southern Ridge and Valley | **-1.0843** | **0.3143** | **-1.6965** | **-0.4622** | **-3.450** | **0.338** | **0.183** | **0.630** | **0.000561** |
| Bioregion: Upper-Northern Piedmont | **-1.4166** | **0.3305** | **-2.0631** | **-0.7655** | **-4.286** | **0.243** | **0.127** | **0.465** | **1.82E-05** |
| Drainage area | **-0.0021** | **0.0011** | **-0.0043** | **0.0000** | **-1.962** | **0.998** | **0.996** | **1.000** | **0.049789** |
| Elevation | **-0.0012** | **0.0004** | **-0.0020** | **-0.0004** | **-2.889** | **0.999** | **0.998** | **1.000** | **0.003863** |
| Clay soils | -0.0144 | 0.0101 | -0.0343 | 0.0055 | -1.421 | 0.986 | 0.966 | 1.005 | 0.155307 |
| Estimated mean depth to water table | **0.0171** | **0.0039** | **0.0095** | **0.0249** | **4.344** | **1.017** | **1.010** | **1.025** | **1.40E-05** |
| Percent Calcium Oxide in lithology | **0.0585** | **0.0090** | **0.0408** | **0.0762** | **6.481** | **1.060** | **1.042** | **1.079** | **9.09E-11** |
| Topographic wetness index | 0.0647 | 0.0428 | -0.0192 | 0.1487 | 1.511 | 1.067 | 0.981 | 1.160 | 0.130751 |
| Uniaxial Compressive Strength (UCS) | 0.0031 | 0.0021 | -0.0009 | 0.0072 | 1.510 | 1.003 | 0.999 | 1.007 | 0.131153 |
| Freshwater withdrawal | **-0.0013** | **0.0005** | **-0.0024** | **-0.0003** | **-2.535** | **0.999** | **0.998** | **1.000** | **0.011242** |
| Density NPDES locations | 0.0661 | 0.0418 | 0.0028 | 0.1801 | 1.581 | 1.068 | 1.003 | 1.197 | 0.113774 |
| Canal/ditch/pipeline | -0.0214 | 0.0143 | -0.0534 | 0.0037 | -1.500 | 0.979 | 0.948 | 1.004 | 0.133506 |
| Open water | **0.1592** | **0.0568** | **0.0580** | **0.2748** | **2.805** | **1.173** | **1.060** | **1.316** | **0.005036** |
| Barren land | **0.1471** | **0.0412** | **0.0684** | **0.2309** | **3.573** | **1.158** | **1.071** | **1.260** | **0.000353** |
| Shrub/scrub | 0.0354 | 0.0207 | -0.0059 | 0.0753 | 1.712 | 1.036 | 0.994 | 1.078 | 0.086834 |
| Grassland/herbaceous | **0.0540** | **0.0200** | **0.0152** | **0.0937** | **2.706** | **1.055** | **1.015** | **1.098** | **0.006819** |
| Development | **0.0375** | **0.0050** | **0.0278** | **0.0475** | **7.452** | **1.038** | **1.028** | **1.049** | **9.22E-14** |
| Agriculture | **0.0197** | **0.0033** | **0.0132** | **0.0262** | **5.921** | **1.020** | **1.013** | **1.027** | **3.20E-09** |
| Wetlands | **0.0371** | **0.0079** | **0.0217** | **0.0528** | **4.689** | **1.038** | **1.022** | **1.054** | **2.74E-06** |

Table S9. Results of Fisher’s Exact Test for the paired gage and Chessie BIBI sites (n = 50) for each of the 12 hydrologic metrics depicting the odds of a degraded macroinvertebrate condition in a flow-altered site.

| Hydrologic Metric | Flow | Observed HM data | | | | |  | Modeled HM data | | | | |
| --- | --- | --- | --- | --- | --- | --- | --- | --- | --- | --- | --- | --- |
|  |  | Stream Condition | |  | Fisher's Exact Test | |  | Stream Condition | |  | Fisher's Exact Test | |
|  |  | Not Degraded | Degraded |  | p-value | Odds Ratio |  | Not Degraded | Degraded |  | p-value | Odds Ratio |
| HF_DUR | Not Altered | 14 | 12 |  | 0.1663 | 2.3 |  | 15 | 9 |  | 0.1713 | 2.2 |
|  | Altered | 8 | 16 |  |  | (0.6-8.6) |  | 11 | 15 |  |  | (0.6-8.2) |
|  |  |  |  |  |  |  |  |  |  |  |  |  |
| HF_REF | Not Altered | 15 | 11 |  | 0.09839 | 2.7 |  | 16 | 10 |  | 0.2571 | 2.2 |
|  | Altered | 8 | 16 |  |  | (0.8-10.1) |  | 10 | 14 |  |  | (0.6-8.1) |
|  |  |  |  |  |  |  |  |  |  |  |  |  |
| HF_MAG | Not Altered | 14 | 12 |  | 0.7775 | 1.4 |  | 16 | 9 |  | 0.1564 | 2.6 |
|  | Altered | 11 | 13 |  |  | (0.4-4.8) |  | 10 | 15 |  |  | (0.7-9.8) |
|  |  |  |  |  |  |  |  |  |  |  |  |  |
| HF_SEA | Not Altered | 14 | 12 |  | 0.272 | 1.9 |  | 16 | 9 |  | 0.1564 | 2.6 |
|  | Altered | 9 | 15 |  |  | (0.5-7.0) |  | 10 | 15 |  |  | (0.7-9.8) |
|  |  |  |  |  |  |  |  |  |  |  |  |  |
| HF_VAR | Not Altered | 14 | 12 |  | 0.4129 | 1.6 |  | 18 | 12 |  | 0.2484 | 2.2 |
|  | Altered | 10 | 14 |  |  | (0.5-5.8) |  | 8 | 12 |  |  | (0.6-8.4) |
|  |  |  |  |  |  |  |  |  |  |  |  |  |
| LF_DUR | Not Altered | 13 | 13 |  | 0.1588 | 2.4 |  | 16 | 8 |  | 0.0546 | 3.1 |
|  | Altered | 7 | 17 |  |  | (0.7-9.3) |  | 10 | 16 |  |  | (0.9-12.0) |
|  |  |  |  |  |  |  |  |  |  |  |  |  |
| LF_FRE | Not Altered | 13 | 13 |  | 0.265 | 2.0 |  | 17 | 8 |  | **0.0465** | 3.7 |
|  | Altered | 8 | 16 |  |  | (0.6-7.4) |  | 9 | 16 |  |  | (1.0-14.4) |

Table S9 continued.

| Hydrologic Metric | Flow | Observed HM data | | | | |  | Modeled HM data | | | | |
| --- | --- | --- | --- | --- | --- | --- | --- | --- | --- | --- | --- | --- |
|  |  | Stream Condition | |  | Fisher's Exact Test | |  | Stream Condition | |  | Fisher's Exact Test | |
|  |  | Not Degraded | Degraded |  | p-value | Odds Ratio |  | Not Degraded | Degraded |  | p-value | Odds Ratio |
| LF_MAG | Not Altered | 14 | 12 |  | 0.775 | 1.4 |  | 17 | 12 |  | 0.3905 | 1.9 |
|  | Altered | 11 | 13 |  |  | (0.4-4.8) |  | 9 | 12 |  |  | (0.5-6.9) |
|  |  |  |  |  |  |  |  |  |  |  |  |  |
| LF_SEA | Not Altered | 14 | 12 |  | 0.272 | 1.9 |  | 16 | 10 |  | 0.2571 | 2.2 |
|  | Altered | 9 | 15 |  |  | (0.5-7.0) |  | 10 | 14 |  |  | (0.6-8.1) |
|  |  |  |  |  |  |  |  |  |  |  |  |  |
| LF_VAR | Not Altered | 14 | 12 |  | 0.4129 | 1.6 |  | 17 | 11 |  | 0.2542 | 2.2 |
|  | Altered | 10 | 14 |  |  | (0.5-5.8) |  | 9 | 13 |  |  | (0.6-8.1) |
|  |  |  |  |  |  |  |  |  |  |  |  |  |
| SKEW | Not Altered | 19 | 7 |  | **0.04378** | 3.7 |  | 20 | 12 |  | 0.0765 | 3.3 |
|  | Altered | 10 | 14 |  |  | (1.0-14.8) |  | 6 | 12 |  |  | (0.9-13.6) |
|  |  |  |  |  |  |  |  |  |  |  |  |  |
| RISES | Not Altered | 13 | 13 |  | 0.5835 | 1.4 |  | 15 | 11 |  | 0.5716 | 1.6 |
|  | Altered | 10 | 14 |  |  | (0.4-5.0) |  | 11 | 13 |  |  | (0.5-5.7) |


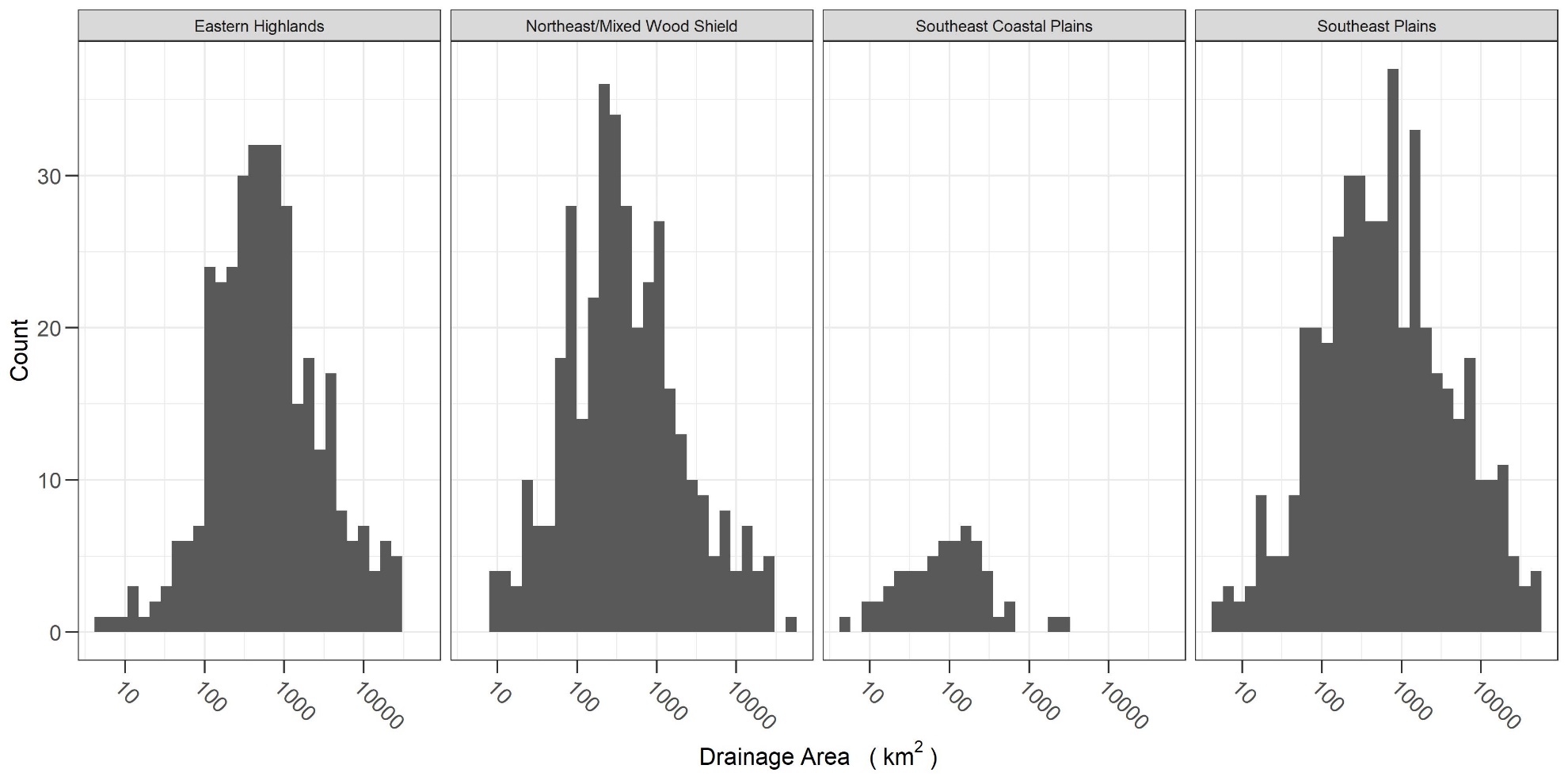


Figure S1. Distribution of upstream drainage area for gages used to build random forest models for each hydrologic metric by aggregated Level III ecoregion.


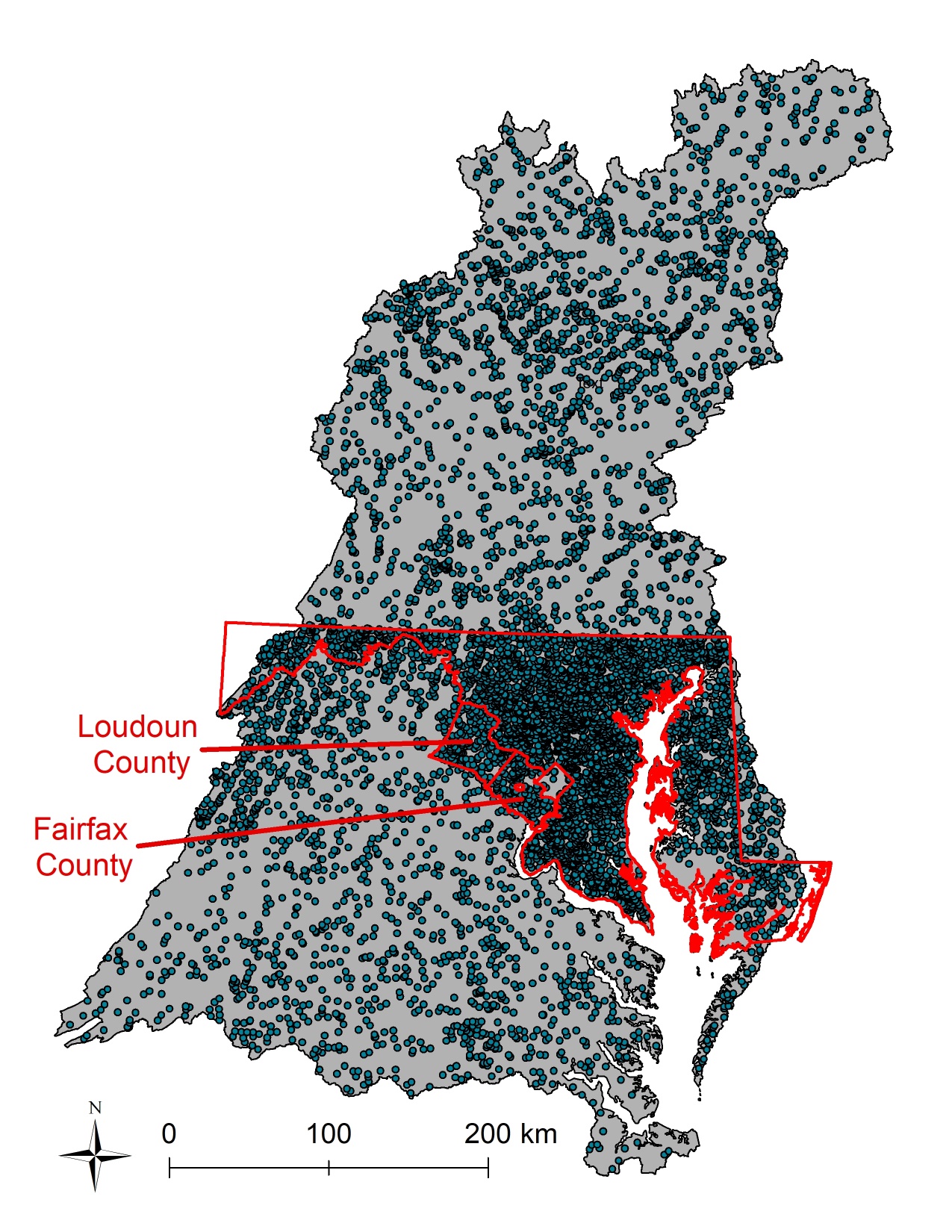


Figure S2. Map showing clustering of Chesapeake Bay Basin-wide Index of Biotic Integrity data for Maryland and Fairfax and Loudoun Counties (border highlighted in red) for Chesapeake Bay Watershed.


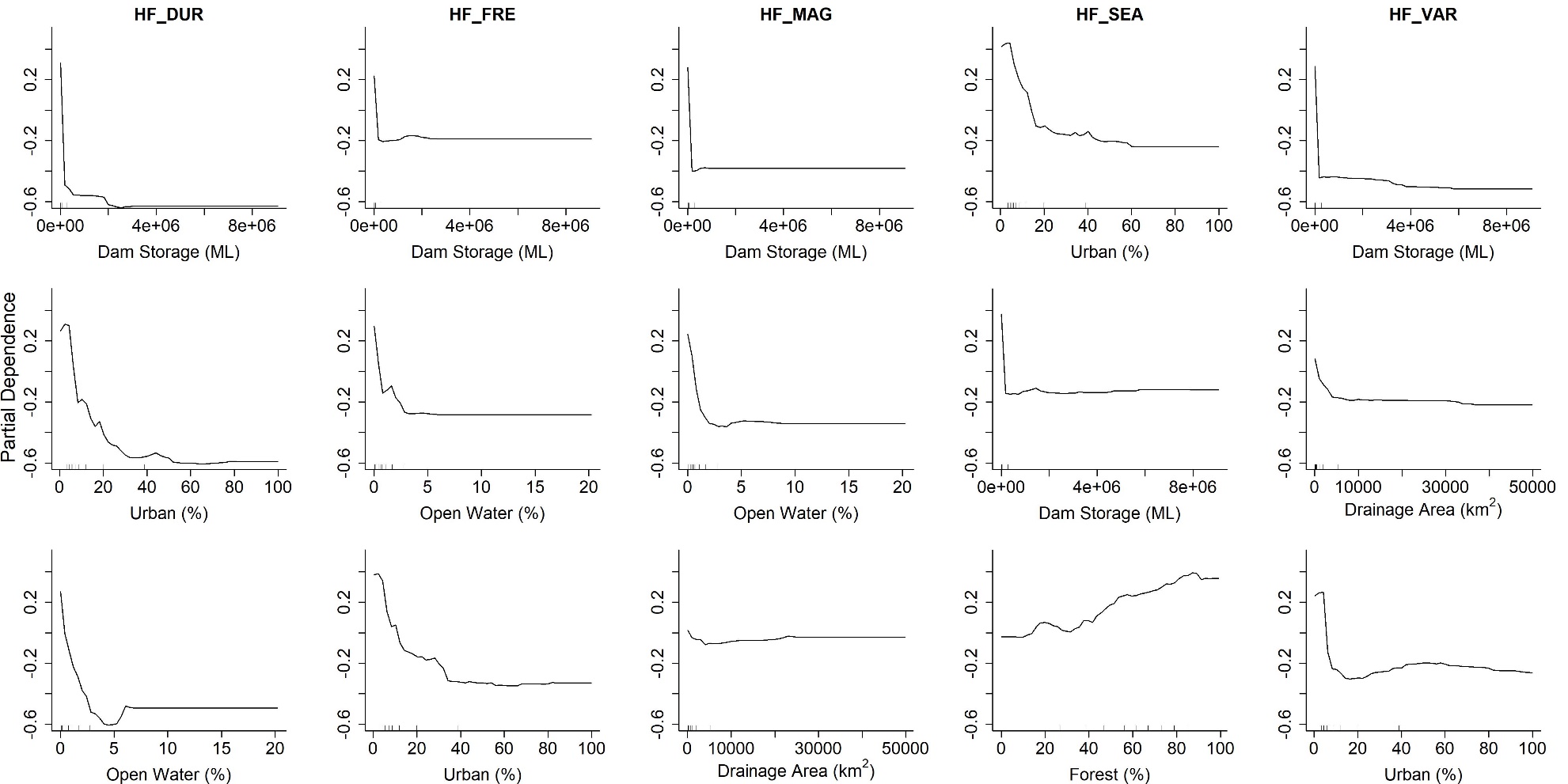
Figure S3. Partial dependence plots of the top three important predictors, from top to bottom, in each random forest model for the five high-flow hydrologic metrics. Partial dependence plots show the marginal effect of the selected predictor on the probability of an Indeterminant flow classification.


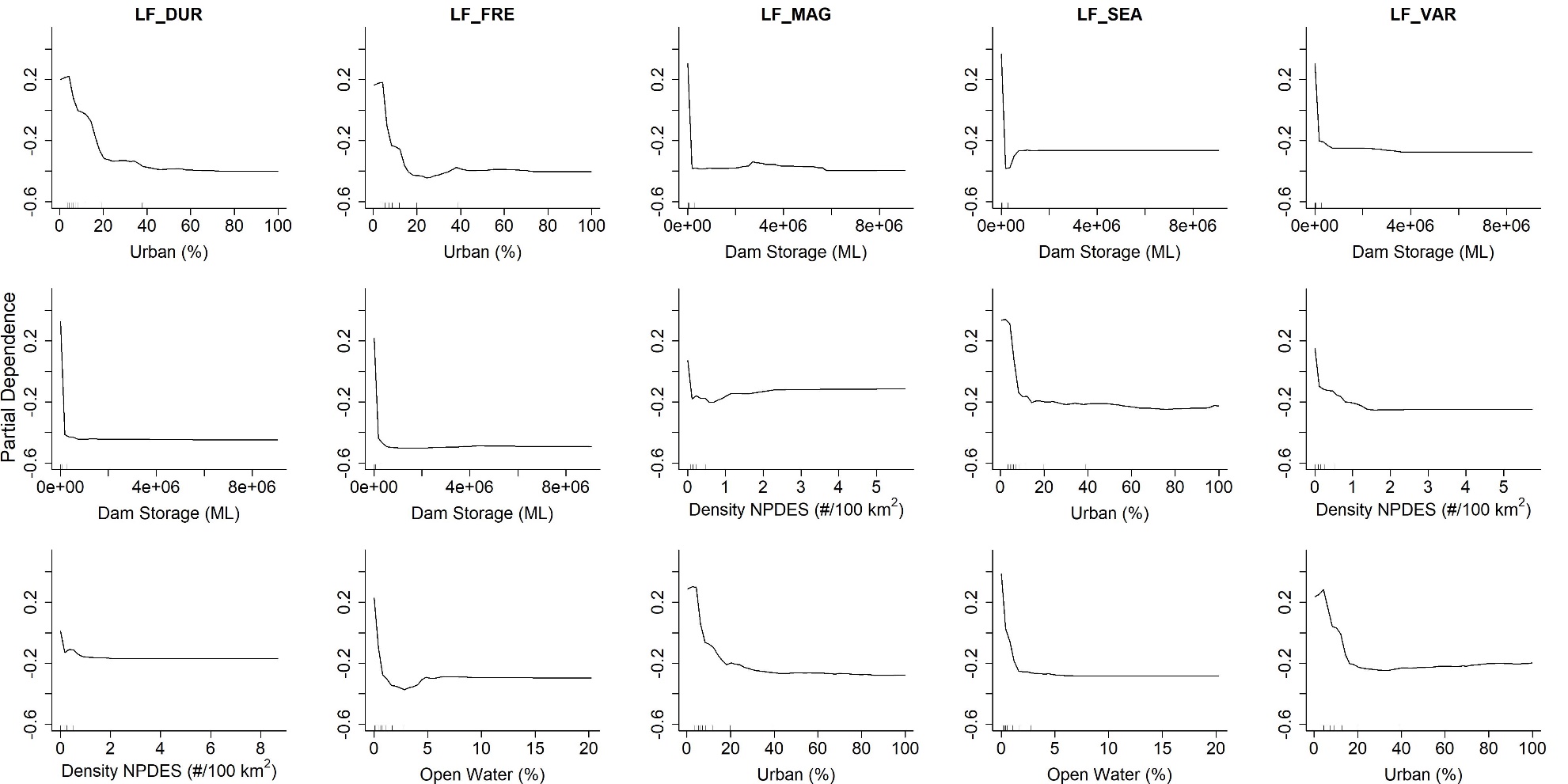


Figure S4. Partial dependence plots of the top three important predictors, from top to bottom, in each random forest model for the five low-flow hydrologic metrics. Partial dependence plots show the marginal effect of the selected predictor on the probability of an Indeterminant flow classification.


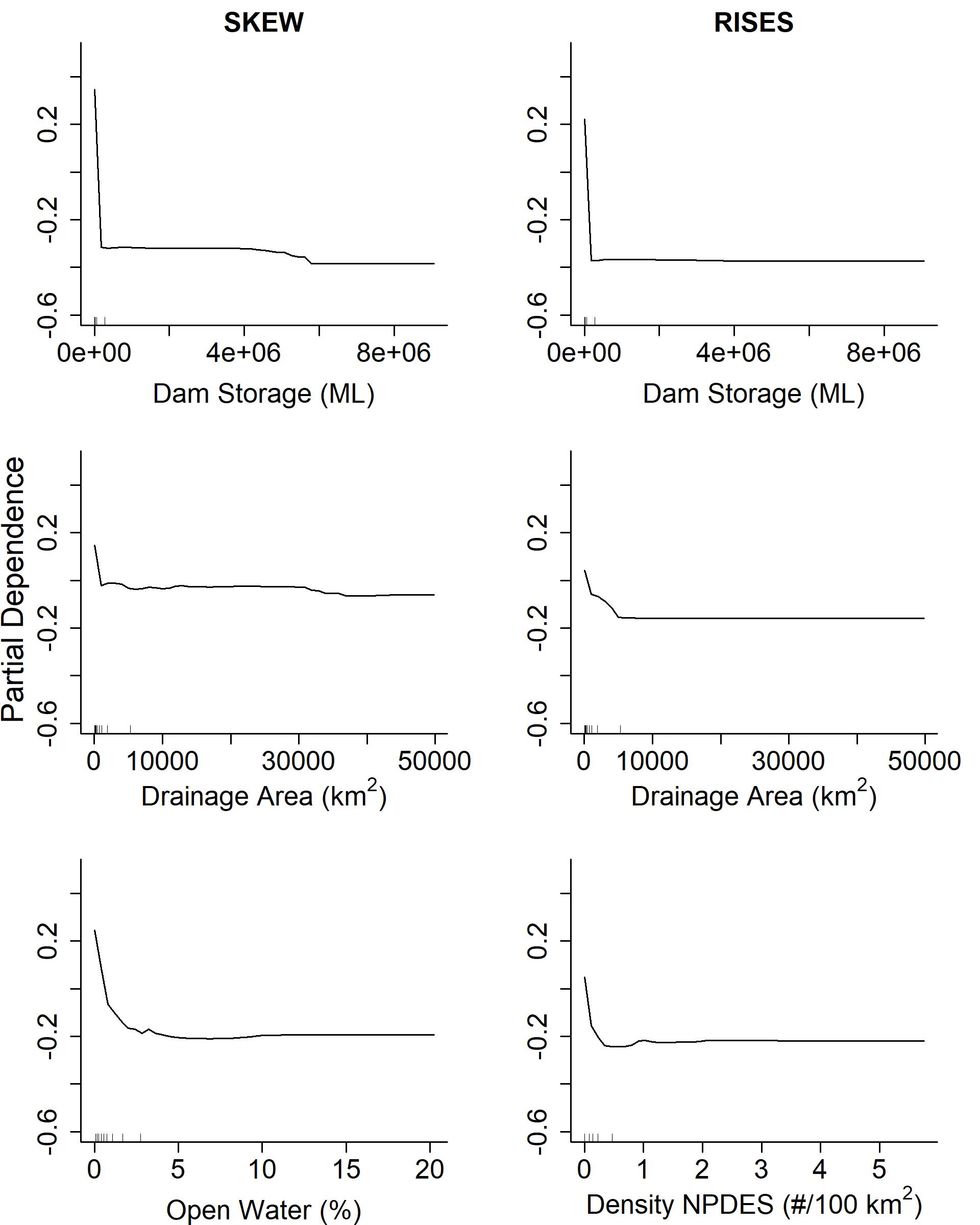


Figure S5. Partial dependence plots of the top three important predictors, from top to bottom, in each random forest model for skew (SKEW) and rises (RISES). Partial dependence plots show the marginal effect of the selected predictor on the probability of an Indeterminant flow classification.


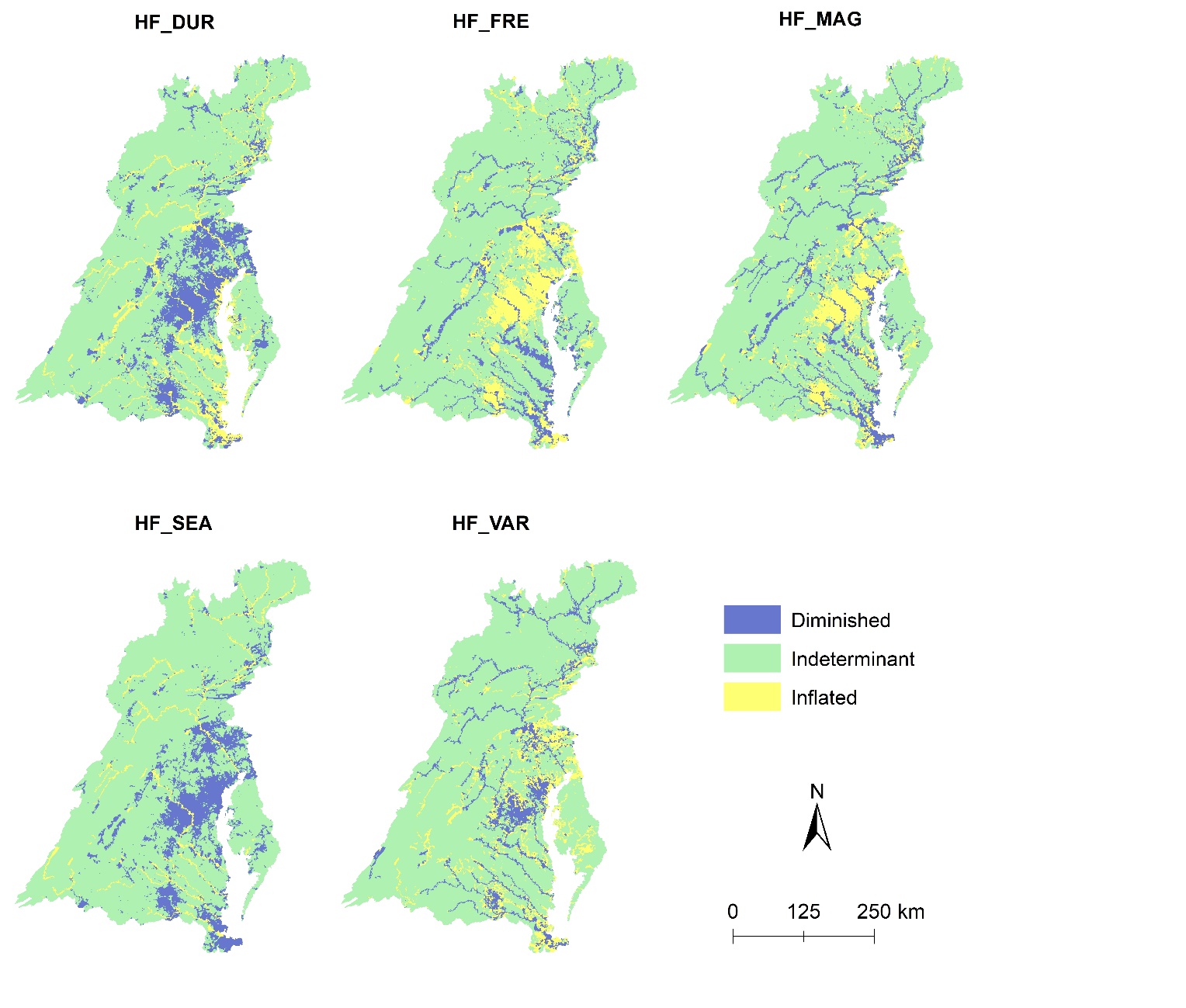


Figure S6. Maps showing spatial distribution of altered flow categories for the five high-flow hydrologic metrics for the Chesapeake Bay watershed. (See Table 1 for hydrologic metric definitions).


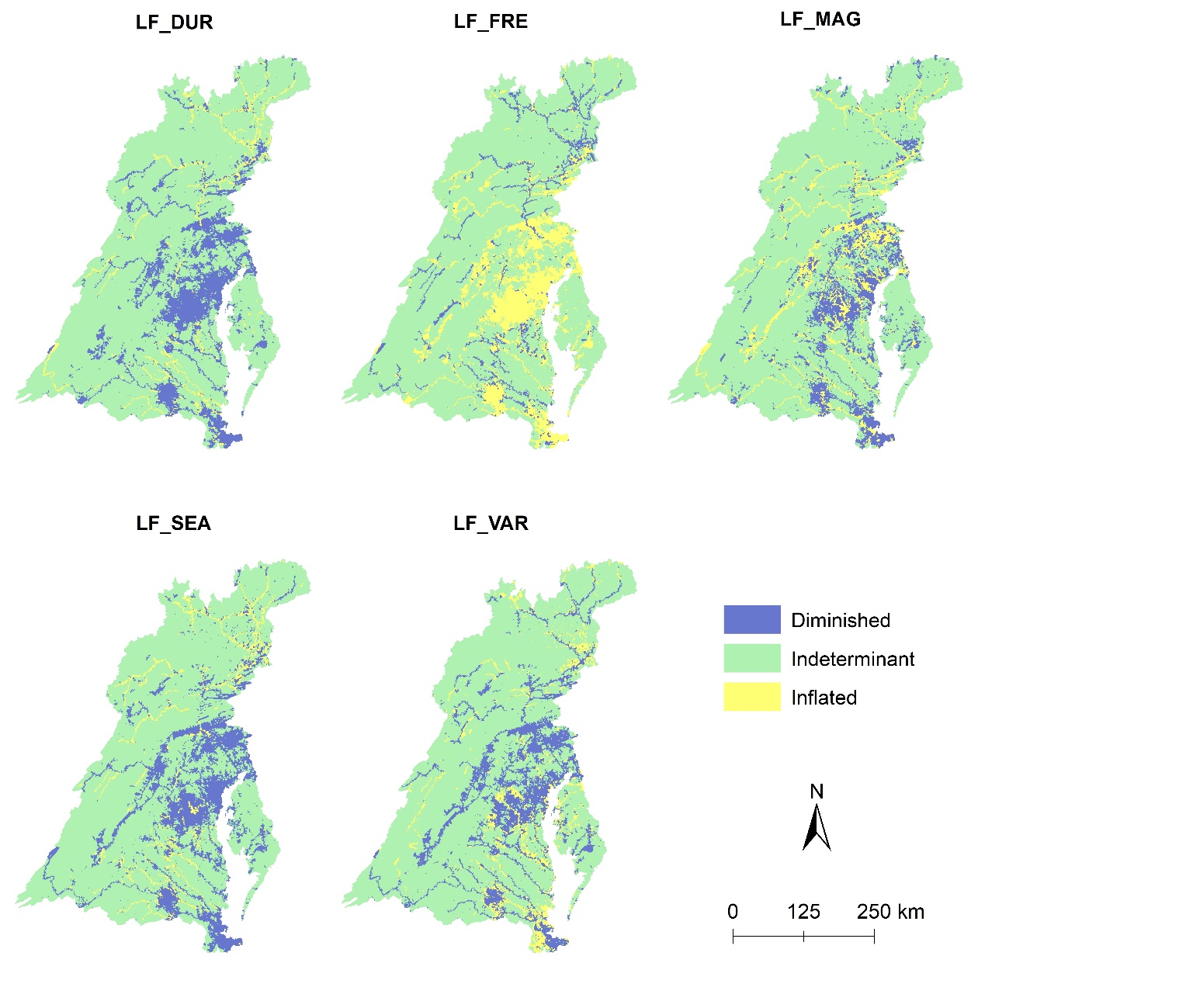


Figure S7. Maps showing spatial distribution of altered flow categories for the five low-flow hydrologic metrics for the Chesapeake Bay watershed. (See Table 1 for hydrologic metric definitions).


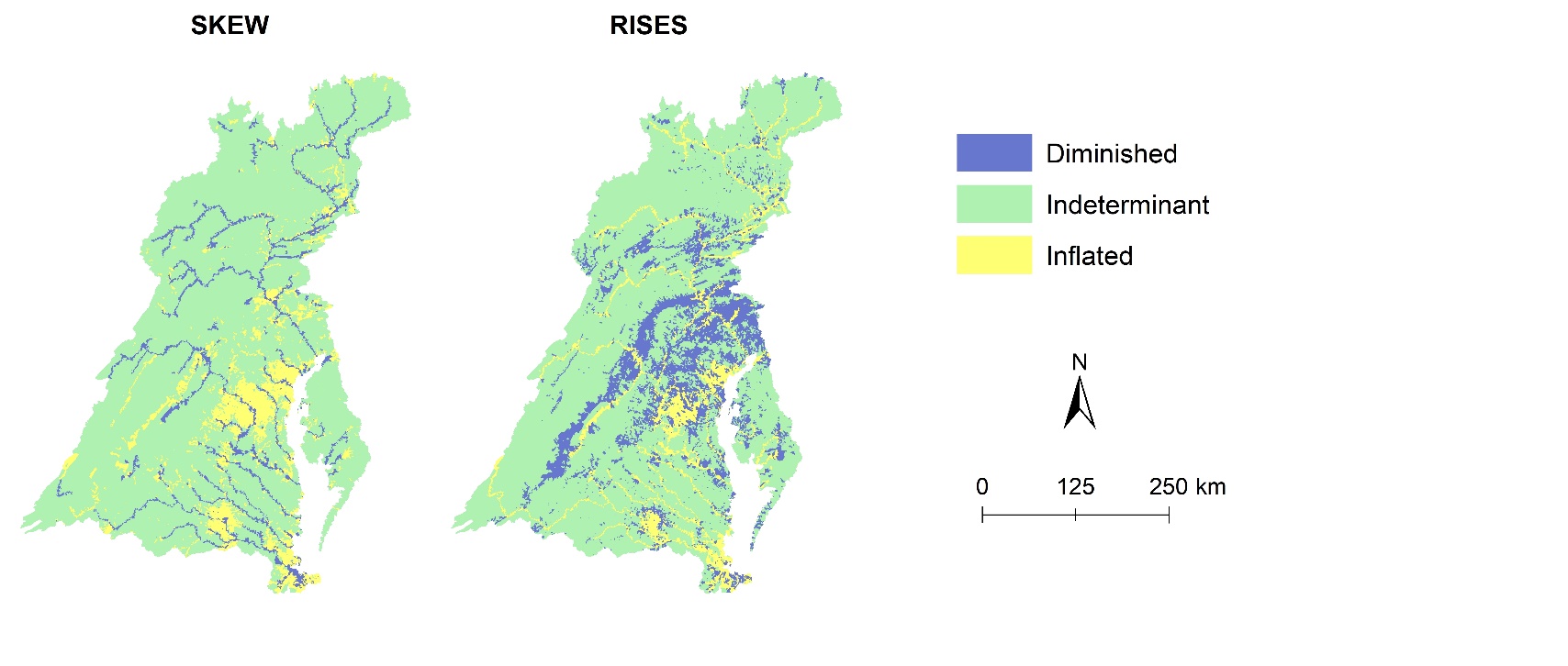


Figure S8. Maps showing spatial distribution of altered flow categories for the two symmetry and stochasticity hydrologic metrics for the Chesapeake Bay watershed. (See Table 1 for hydrologic metric definitions).


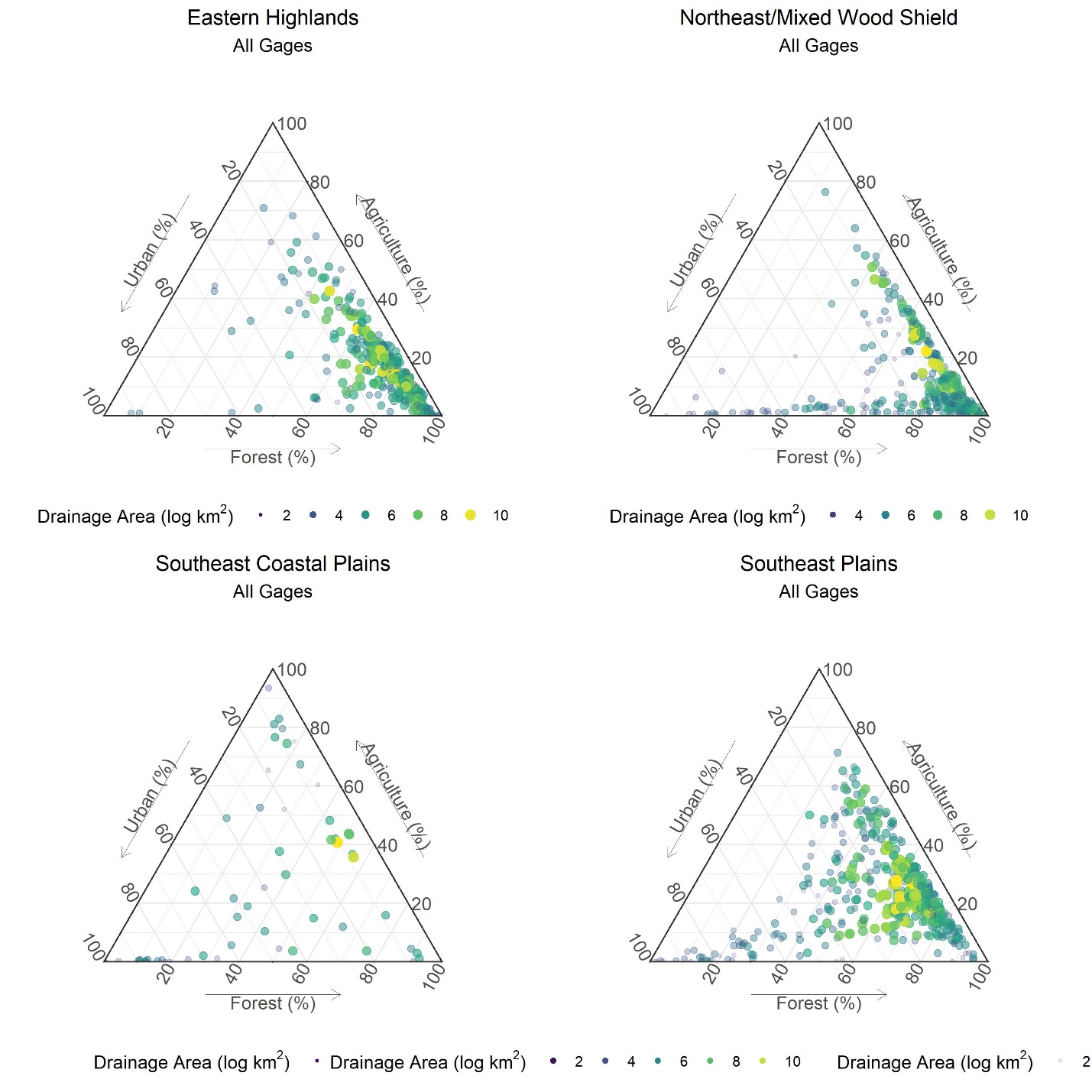


Figure S9. Ternary plot showing gages used in random forest models by urban, agriculture, and forest covers. Size and color of circle represents drainage size. Figures created using the ggtern R package and function (Hamilton and Ferry, 2018).


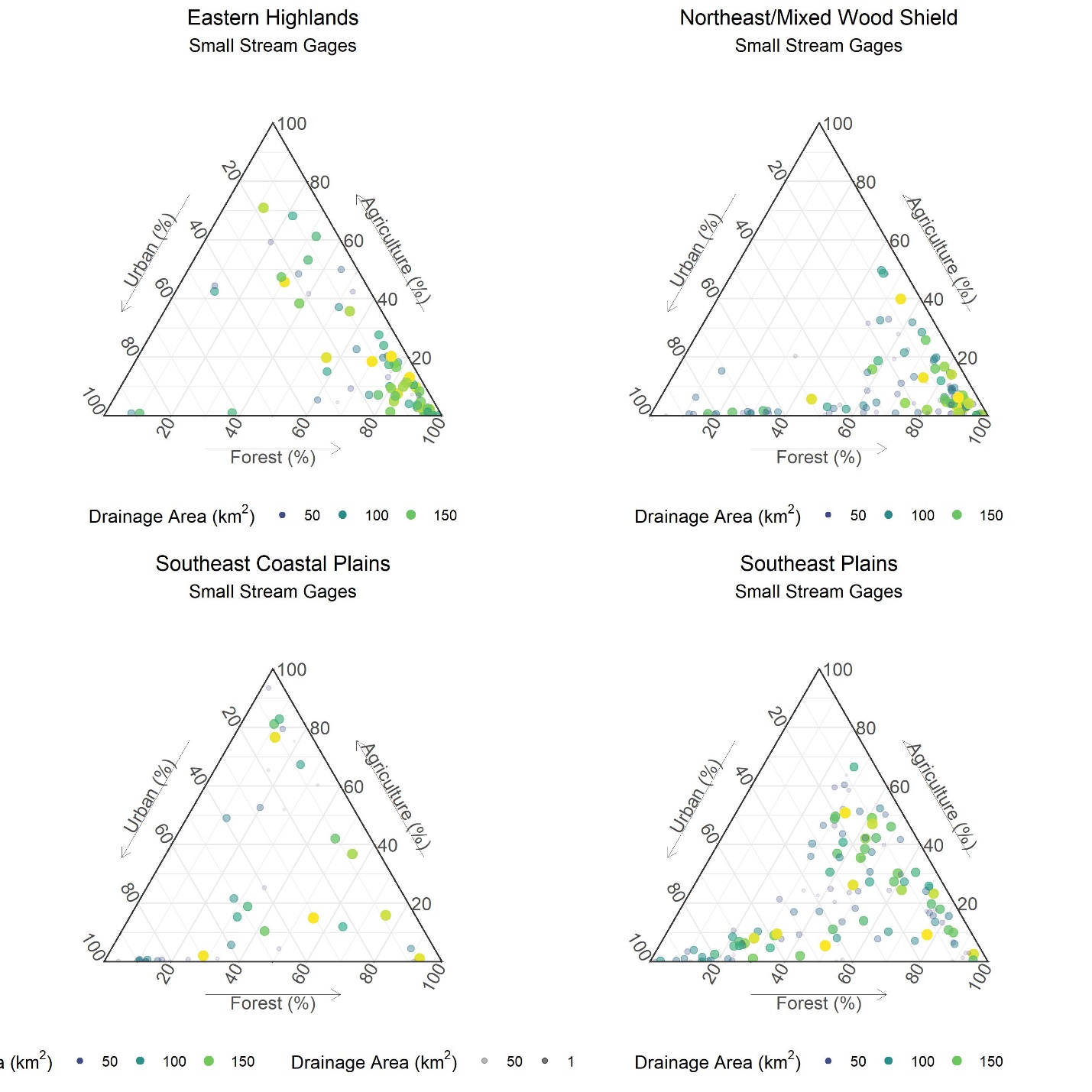
Figure S10. Ternary plot showing gages on small stream reaches (< 200 km^2^ upstream drainage) by urban, agriculture, and forest covers. Size and color of circle represents drainage size. Figures created using the ggtern R package and function (Hamilton and Ferry, 2018).


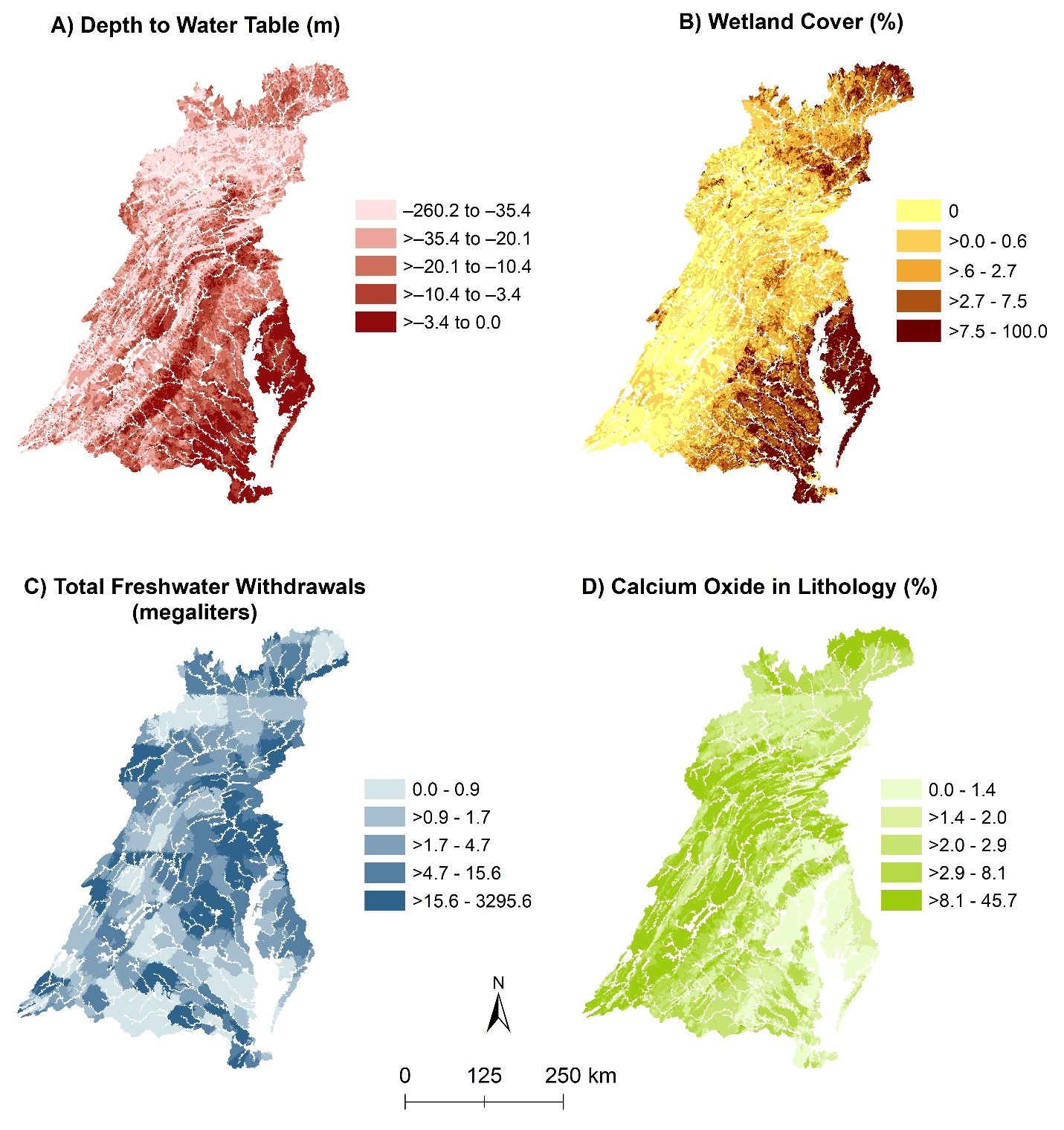
Figure S11. Maps showing spatial representation of A) the depth to water table, B) percent wetland cover, C) total freshwater withdrawals, and D) calcium oxide in lithology for catchments with upstream drainages < 200 km^2^ (small streams) for the Chesapeake Bay watershed.


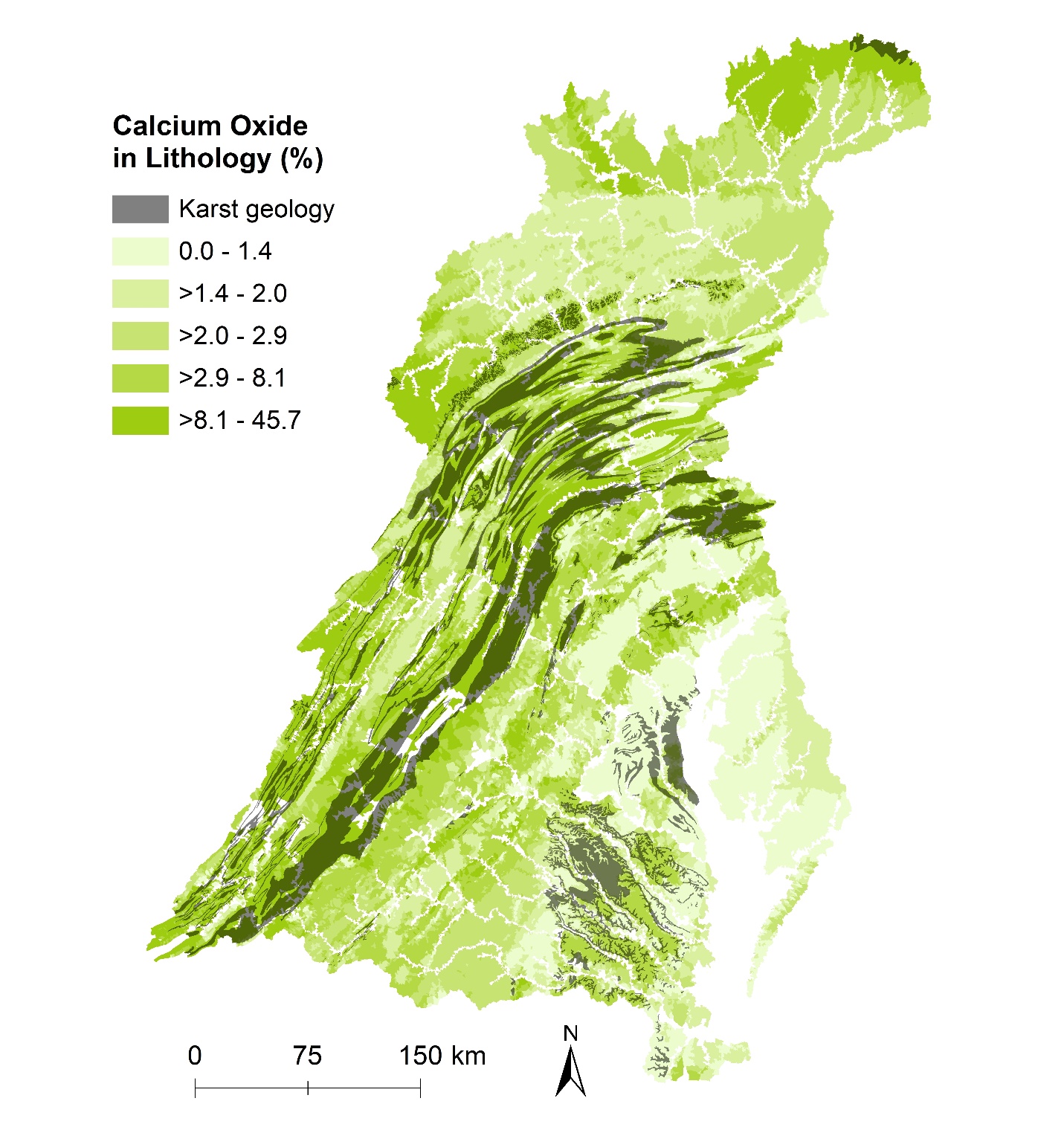


Figure S12. Map showing calcium oxide in lithology with karst geology overlain for the Chesapeake Bay watershed.

**References for supplements**

Hamilton NE, Ferry M (2018). ggtern: Ternary Diagrams Using ggplot2. Journal of Statistical Software, Code Snippets, 87(3), 1-17. doi: 10.18637/jss.v087.c03 (URL: https://doi.org/10.18637/jss.v087.c03).
